# Isoflurane and Surgery Aggravate APOE4-Dependent Lipid Dysregulation, Neural Dysfunction, and Neurological Impairment in Male Mice

**DOI:** 10.3390/cells15212038

**Published:** 2026-11-09

**Authors:** Yun Li, Yuanyuan Ji, Cosar Uzun, Syed Taufiqul Islam, Meigeng Hu, Dan Zhao, Yaping Li, Hangnoh Lee, Zihui Wang, Hui Li, Jace W. Jones, Shaolin Liu, Junfang Wu

**Affiliations:** 1 Department of Anesthesiology and Center for Shock, Trauma and Anesthesiology Research (STAR), University of Maryland School of Medicine, Baltimore, MD 21201, USA; 2 Department of Pharmaceutical Sciences, University of Maryland School of Pharmacy, Baltimore, MD 21201, USA; 3 Center for Neurological Disease Research, University of Georgia College of Veterinary Medicine, Athens, GA 30602, USA; 4 Department of Physiology and Pharmacology, University of Georgia College of Veterinary Medicine, Athens, GA 30602, USA; 5 Department of Biomedical Sciences, University of Georgia College of Veterinary Medicine, Athens, GA 30602, USA; 6 Department of Medicine, University of Maryland School of Medicine, Baltimore, MD 21201, USA; 7 University of Maryland-Medicine Institute for Neuroscience Discovery (UM-MIND), University of Maryland School of Medicine, Baltimore, MD 21201, USA

**Keywords:** isoflurane: APOE4, lipidomics, brain circuitry, anesthesia, neurological dysfunction

## Abstract

Perioperative exposure to the volatile anesthetic isoflurane (ISO) during surgery (OP) has been associated with cognitive and olfactory deficits and may increase the risk of Alzheimer’s disease (AD). Apolipoprotein E4 (APOE4), the strongest genetic risk factor for AD, contributes to disease pathogenesis through disrupted lipid homeostasis. However, whether ISO/OP interacts with APOE genotype to influence neurological vulnerability remains unclear. Young adult, presymptomatic humanized APOE4 and APOE3 knock-in mice underwent laparotomy under 2 h of ISO anesthesia. Microglia and astrocytes were isolated from the olfactory bulb (OB) and hippocampus (HI) for lipidomic profiling. Awake in vivo OB recordings assessed neuronal excitability and network activity, while ex vivo patch-clamp recordings assessed synaptic transmission in the OB and HI. On day 7, APOE4/ISO/OP glia showed greater lipid perturbations than APOE3/ISO/OP glia. In vivo recordings revealed genotype-dependent alterations in OB excitability and network operation, while patch-clamp recordings identified altered excitatory and inhibitory synaptic transmission in the OB and HI. APOE4/ISO/OP mice exhibited persistent odor memory deficits, transient olfactory sensitivity loss, early motor impairment, and delayed cognitive dysfunction. OB RNA sequencing identified alterations in calcium transport and metabolic pathways in APOE4 male mice at 90 days following exposure. Together, these findings demonstrate APOE4-dependent vulnerability to ISO/OP, characterized by glial lipid dysregulation, neural circuit dysfunction, and neurological impairment, and support a potential mechanistic contribution of altered lipid homeostasis.

## 1. Introduction

General anesthesia (GA) is essential for modern surgery, but perioperative exposure to volatile anesthetics and surgical stress may contribute to postoperative neurocognitive dysfunction (PND), characterized by impairments in memory, attention, and executive function [1,2,3]. Anesthesia and surgery can also induce olfactory dysfunction (OD), and emerging evidence suggests that postoperative OD may precede or associate with later cognitive decline [4,5,6,7]. Beyond its impact on quality of life, OD is increasingly recognized as an early marker of neurodegenerative disease, including Alzheimer’s disease (AD) [8,9,10]. Longitudinal studies indicate that OD can precede cognitive decline by years or decades [11,12], potentially through olfactory bulb (OB)-linked propagation of pathogenic proteins and disrupted connectivity with limbic memory circuits [6,10].

Preclinical studies support anesthesia-associated neurotoxicity, including Tau phosphorylation after repeated sevoflurane exposure and long-term memory impairment after isoflurane (ISO) exposure in rodents [13,14,15,16]. However, how transient perioperative insults might interact with genetic risk to promote later AD-related vulnerability remains unclear. The apolipoprotein E4 (APOE4) allele is the strongest common genetic risk factor for sporadic AD, conferring an approximately threefold increased risk in heterozygotes and a 10- to 15-fold increased risk in homozygotes compared with non-carriers [17,18]. Approximately 25–30% of U.S. adults carry at least one APOE4 allele [19,20]. Clinical and preclinical studies suggest that APOE4 may increase susceptibility to PND, but direct apolipoprotein APOE3 versus APOE4 comparisons remain limited [21,22,23,24,25]. Humanized E4 knock-in (KI) mice recapitulate key aspects of E4-driven vulnerability without overexpressing mutant amyloid or tau, providing a genetically precise and translationally relevant platform for studying anesthesia-related neurotoxicity [26,27]. APOE4 is linked to age-dependent changes in glial lipid metabolism, synaptic integrity, and network excitability, including lipid droplet accumulation and impaired glial support of neuronal function [28,29]. Moreover, APOE4-associated disruption of astrocytic fatty acid metabolism, lipid droplet regulation, and neuron–astrocyte metabolic coupling can compromise neuronal metabolic support and synaptic homeostasis [30,31]. These established relationships provide a mechanistic basis for hypothesizing that perioperative stress may exacerbate APOE4-dependent glial lipid dysregulation, potentially contributing to neuronal circuit dysfunction and subsequent neurological impairment. Yet glial lipid dysregulation remains underexplored in PND models, and whether perioperative exposure interacts with APOE genotype to shape postoperative neurological risk is unknown.

Therefore, we hypothesized that APOE4 interacts with ISO exposure and surgical stress to exacerbate neurological disruption. To test this, presymptomatic young adult APOE3 and APOE4 knock-in mice underwent laparotomy with 2 h of ISO anesthesia. Using integrated lipidomic, electrophysiological, behavioral, and transcriptomic analyses, we identified APOE4-specific vulnerabilities marked by early glial lipid dysregulation, olfactory circuit dysfunction, and persistent sensory deficits that preceded later cognitive impairment. These findings establish olfactory dysfunction as an early, accessible marker of APOE4 susceptibility, highlighting a window for intervention to mitigate anesthesia- and age-related cognitive decline.

## 2. Methods

### 2.1. Mouse General Anesthesia and Operation

All experimental procedures were approved by the Institutional Animal Care and Use Committees of the University of Maryland School of Medicine and the University of Georgia. Homozygous humanized APOE4 KI [B6(SJL)-Apoetm1.1(^APOE*4^)Adiuj/J, stock no. 027894] and APOE3 KI [B6.Cg-Apoeem2^(APOE*)^Adiuj/J, stock no. 029018] mice (Mus musculus; male; 10–14 weeks old; 20–25 g) were obtained from The Jackson Laboratory (Bar Harbor, ME, USA). Mice were subjected to a perioperative paradigm consisting of 2 h ISO anesthesia with laparotomy operation (OP) to model surgical stress, as detailed in Supplemental Materials. Sham controls remained in their home cages and were exposed to room air for 2 h to better model clinically relevant baseline conditions. We present this article in accordance with the ARRIVE 2.0 reporting guidance, with the full checklist included in Supplemental Materials [32]. This 2 h ISO/OP paradigm was used for behavioral testing, lipidomic profiling, and RNA sequencing. Because electrophysiological recordings required modality-specific preparations, anesthetic delivery and surgical procedures differed across cohorts and are described separately below.

### 2.2. Adult Cell Isolation

On day 7 after ISO/OP, mice were perfused with 50 mL of ice-cold saline. OB and hippocampus (HI) tissue from three mice of the same genotype and treatment were pooled per biological replicate. CD11b^+^ microglia and ACSA2^+^ astrocytes were isolated by magnetic-activated cell sorting according to the manufacturer’s protocol (Miltenyi Biotec, Bergisch Gladbach, Germany).

### 2.3. Lipidomics

Lipidomic analysis of astrocyte- and microglia-enriched samples was performed as previously described [33,34]. Data acquisition and processing was performed using Thermo Xcalibur 4.2 software (Thermo Fisher Scientific, Waltham, MA, USA), with additional data processing using TraceFinder 5.1 (Thermo Fisher Scientific, Waltham, MA, USA). Subsequent statistical analyses and data visualization were performed using GraphPad Prism 10 (GraphPad Software, Boston, MA, USA) and MetaboAnalyst 6.0 [35]. In brief, partial least squares discriminant analysis (PLS-DA) was performed using MetaboAnalyst 6.0 to visualize differences in lipid profiles among experimental groups. Pairwise differential abundance analyses were also conducted using MetaboAnalyst 6.0, with *p*-values adjusted for multiple comparisons using the Benjamini–Hochberg procedure. Lipids with false discovery rate (FDR)-adjusted *p* ≤ 0.05 were considered statistically significant. Volcano plots were generated using the EnhancedVolcano R package [36], displaying nominal *p*-values while highlighting lipids meeting the FDR-adjusted significance threshold. Adjusted *p*-values for individual lipid species are provided in the Supplementary Data. Heatmaps were generated for exploratory visualisation of sample clustering and lipid abundance patterns using the 30 lipids with the lowest nominal *p*-values from Student’s *t*-tests in each comparison. To evaluate analytical reproducibility, coefficients of variation (CVs) were calculated for three isotope-labelled internal standards representing cardiolipins (CLs), hexosylceramides (HexCers), and triglycerides (TGs) across experimental groups (Table S1). These internal standards generally exhibited lower CVs than the corresponding endogenous lipid species (Table S2). Complete differential abundance results, including nominal and FDR-adjusted *p*-values for all lipid species across pairwise comparisons, are provided in Supplementary Materials 3 (Excel File).

### 2.4. In Vivo Electrophysiological Recording and Data Analysis

Surgical preparations for in vivo electrophysiological recordings and data analysis were performed as previously described with modifications [7,36,37]. Mice underwent cranial headplate implantation and neural-probe access surgery for awake recordings, followed by recovery before ISO exposure. No laparotomy or other non-cranial surgery was performed in this cohort at the time of ISO exposure. To assess the short-term and long-term effects of ISO and surgery, recordings were performed 24 h or 7 days after the GA cessation, respectively. Spontaneous neuronal excitability and network activities were recorded as described previously [36,37], with the modification that a 32-channel neural probe (60 μm shank width, 15 μm shank thickness, and 50 μm inter-channel distance) was inserted into the MCL on the medial side of each OB.

Spikes were sorted from the raw data with Offline Sorter V4 software (Plexon). Individual units were separated via principal component analysis based on waveform parameters and multidimensional cluster criteria. Spike and local field potential (LFP) data were further analyzed with NeuroExplorer V5.204 (Nex Technologies).

Instantaneous spike frequencies (ISFs), calculated from inter-spike intervals of individual units, were averaged across units to represent each recording, and multiple recordings from each animal were then averaged to obtain an animal-level ISF. To compare neuronal excitability among groups, cumulative probability distributions of ISF were averaged across animals within each group.

The same hierarchical summarizing strategy (recording → animal → group) was applied to the LFP data. The power spectrum density (PSD) of LFP signals ranging from 0.1 to 200 Hz was measured to quantify the strength of oscillatory activity, which arises from interactions between excitatory and inhibitory neurons and therefore reflects network operation [37,38,39]. Analyses covered the theta (0–12 Hz), beta (12–30 Hz), and gamma (low gamma, 30–60; high gamma, 60–100 Hz) frequency bands.

Spike and LFP data were presented as mean ± SEM and plotted with Excel and OriginPro 2024, with the number of animals as the sample size (n) for each group. Statistical analyses were performed using custom Python (version 3.15.2) scripts (pandas version 3.1.1, NumPy version 2.5.4, and SciPy version 1.18.1). The cumulative probability (CP) of spontaneous spike frequencies and the PSD of LFP oscillations were analyzed independently at each frequency bin. Pairwise comparisons between groups were performed using Welch’s independent-samples *t*-test, which does not assume equal variances. To control the false discovery rate (FDR) across multiple frequency-wise comparisons, *p*-values were adjusted using the Benjamini–Hochberg (BH) FDR method. As a complementary non-parametric approach, an empirical permutation test was also performed, in which animals were randomly reassigned between groups while maintaining sample sizes, with permutations applied at each frequency bin. Frequency bins with an adjusted *p* < 0.05 were considered statistically significant. For frequency ranges identified as statistically significant by the above methods, group comparisons were additionally performed on the animal-level data using a two-sample *t*-test and presented as bar graphs.

### 2.5. Patch Clamp Recording in Brain Slices and Data Analysis

For patch-clamp experiments, mice were assigned to Sham/OP or ISO/OP cohorts. Both cohorts underwent laparotomy under ISO delivered in room air. Sham/OP mice were allowed to recover shortly after completion of the laparotomy, whereas ISO/OP mice were maintained under 2% ISO to complete a total exposure time of 2 h. This design controlled for laparotomy while testing the contribution of prolonged ISO exposure to synaptic changes. Patch-clamp recordings were performed 7 days after GA cessation as a follow-up experiment to further characterize the ISO-associated electrophysiological alterations observed in vivo.

Acute OB and brain slices containing HI (350 μm thick) were prepared from APOE3 or APOE4 mice as previously described [40,41]. Cumulative probability distribution plots of sEPSCs or sIPSCs were created in Origin 2024. Pairwise comparisons were performed using the non-parametric Kolmogorov–Smirnov test. Two-way ANOVA was used to analyze membrane resistance, resting membrane potential, and current injection-evoked spikes. Notably, *p* ˂ 0.05 was considered statistically significant. Patch pipettes (5–7 MΩ) were pulled from thin-walled glass capillaries with filament (Sutter Instrument, Novato, CA). The internal solution contained (in mM): 117 K-gluconate, 10 KCl, 10 HEPES, 4 EGTA, 12 KOH, 0.5 CaCl_2_, 3 Mg-ATP, 0.3 Na_2_-GTP, and 7 Na_2_-phosphocreatine (pH adjusted to 7.26, 290 mOsm). Automatic detection of spontaneous excitatory or inhibitory postsynaptic currents (sEPSCs or sIPSCs) was performed as previously described with Wdetecta (https://hlab.stanford.edu/wdetecta.php, accessed on 20 December 2024) followed by manual checks to ensure accuracy [40]. Instantaneous sEPSC or sIPSC frequencies were then calculated from their time-stamp data generated from Wdetecta detection.

### 2.6. Assessment of Neurological Function

Behaviors were assessed longitudinally for 88 days by investigators blinded to genotype. Treatment allocation could not be blinded because of the visible surgical incision (Supplementary Figure S1). Groups compared include APOE3/Sham, APOE4/Sham, APOE3/ISO/OP, and APOE4/ISO/OP, with units being a single mouse. Detailed methods and specific tests can be found in Supplementary Materials.

### 2.7. RNA Extraction and Bulk RNA Sequencing (RNAseq)

At 90 days after ISO/OP, mice were perfused with 50 mL of ice-cold saline, and total RNA was isolated from OB and HI using the miRNeasy kit (Qiagen, Cat. No. 217084). Poly(A)-enriched libraries were prepared using Novogene and sequenced as 150 bp paired-end reads on an Illumina NovaSeq 6000 (Illumina, Inc., San Diego, CA, USA).

### 2.8. Statistical Analysis

Data are reported as mean (SEM), with individual values shown where applicable. Normality was assessed using the Shapiro–Wilk test. Electrophysiological and behavioral data were analyzed using ANOVA-based approaches, whereas molecular, transcriptomic, and lipidomic datasets used assay-specific methods. Statistical models, post hoc tests, multiple-testing corrections, sample sizes, and significance thresholds are reported in the relevant Methods subsections and figure legends. Additional design and inclusion details are provided in the Supplemental Information. Unless otherwise specified, *p* < 0.05 was considered significant.

Full details on methods are provided in the Supplementary Data.

## 3. Results

### 3.1. APOE4 Microglia and Astrocytes Show Heightened Lipid Disruption Post-ISO/OP

To investigate the effects of ISO/OP on lipid composition, young adult APOE4 and APOE3 mice were exposed to 2 h of ISO/OP followed by a 7-day recovery period. The OB and HI were subsequently dissected, and microglia and astrocytes were isolated for lipidomic analysis. This approach is particularly relevant because the effects of APOE4 on lipid metabolism are thought to be mediated primarily through glial cells, where it disrupts membrane composition, lipid trafficking, and inflammatory signaling. We first examined genotype-dependent differences by directly comparing APOE4 with APOE3 mice following ISO/OP exposure. In OB astrocytes, PCA and differential analysis revealed genotype-dependent alterations in phosphatidylcholines (PC), phosphatidylethanolamine (PE), and Cer species (Figure 1A–C, *n* = 3 samples/9 mice/group; each sample was pooled from three mice). OB microglia showed similarly robust differences, with shifts across multiple lipid classes and subtypes, including reductions in several triglyceride (TG) species in APOE4/ISO/OP mice compared with APOE3/ISO/OP mice (Figure 1D–F). Although HI astrocytes demonstrated partial overlap between APOE3 and APOE4 groups, distinct alterations in sphingomyelin (SM) and phosphatidylethanolamine (PE) species were observed (Figure 1G–I). In contrast, HI microglia displayed clear separation between APOE4/ISO/OP and APOE3/ISO/OP, with elevated PE and SM emerging as distinctive features of the APOE4 lipid profile (Figure 1J–L).

We next sought to isolate the effects of ISO/OP in a specific genotype. In the OB (Supplementary Figure S2A–F), PCA revealed clear separation between APOE4/ISO/OP and APOE4/Sham groups in both astrocytes and microglia. Differential expression analysis highlighted cell type-specific differences: astrocytes displayed increases in SM, lysophosphatidylcholine (LPC), and lysophosphatidylethanolamine (LPE) species, accompanied by a pronounced reduction in PE, whereas microglia showed reduced PE, with concomitant upregulation of SM and LPE species. In the HI, both astrocytes and microglia exhibited distinct clustering according to treatment (Supplementary Figure S2G–L). Astrocytes showed decreased PE while displaying marked elevations in SM, FA, LPC, and LPE. Similarly, microglia also showed an overall decrease in PE lipids, while FA, LPC, and SM types were elevated after ISO/OP. Comparable disruptions were also detected in APOE3 astrocytes and microglia at 7 d after ISO/OP (Supplementary Figure S3), underscoring the broad impact of anesthesia on glial lipid metabolism. While baseline differences between APOE3 and APOE4 were detectable (Supplementary Figure S4), the combined analysis of lipid classes and subtypes suggested that APOE genotype and ISO/OP exposure jointly influence glial lipid dysregulation. In summary, ISO/OP drives region- and genotype-dependent disruptions to lipid metabolism in both astrocytes and microglia, with APOE4 glia showing more pronounced alterations than glia from APOE3. These distinct cellular signatures underscore glial lipid metabolism as a key node of vulnerability and set the stage for linking such changes to functional outcomes in later analyses.

### 3.2. APOE4 Impairs Neuronal Excitability in the OB of Awake Mice After ISO Exposure

Lipids modulate ion channels, receptors, and neuronal excitability [42,43]. Next, we asked whether ISO-associated glial lipid alterations were accompanied by changes in OB excitability. We performed in vivo recordings from the mitral cell layer using 32-channel linear probes in awake, head-fixed mice on days 1 and 7 after 2 h of ISO exposure (Supplementary Figure S5). Spontaneous spiking and LFP oscillatory activity were recorded across channels to assess neuronal excitability and network function.

On day 1 after exposure (Figure 2A,B), ISO increased mitral cell spontaneous spiking in the frequency range 41–375 Hz (Figure 2(Ba)) in APOE3 mice compared with sham controls (*n* = 3 ISO mice with six recordings; *n* = 3 sham mice with seven recordings), indicating hyperexcitability. In contrast, ISO reduced spiking in the frequency range 106–220 Hz (Figure 2(Bb)) in APOE4 mice compared with APOE4 sham controls (*n* = 3 mice with six recordings per group), with no baseline difference between APOE3 and APOE4 sham groups. These effects are statistically significant when the number of animals (both line and bar graphs) is used as the sample size per group. Thus, ISO produced opposing genotype-dependent effects on mitral cell excitability on day 1.

These ISO effects on APOE4 mice persisted on day 7 (Figure 2C,D). Although no statistically significant ISO effects on APOE3 mice or between APOE3 and APOE4 sham groups at the animal level on day 7 (Figure 2C) were detected, ISO reduced spiking in the frequency range 79–359 Hz (Figure 2D) in APOE4 mice compared with APOE4 sham controls (*n* = 3 ISO mice with eight recordings; *n* = 3 sham mice with six recordings), suggesting a persistent genotype-specific response to ISO. Due to the small number of animals in each group, future studies with larger cohorts are therefore needed to confirm these preliminary findings.

To determine whether ISO effects on neuronal excitability changed over time, we compared spiking frequency between post-ISO days 1 and 7 within each genotype (Supplementary Figure S6). This analysis showed no difference in spiking frequency in sham or ISO-exposed groups of APOE3 mice between day 7 and day 1 (Figure S6A), suggesting no lasting ISO effects on neuronal excitability in this genotype. In contrast, ISO-exposed (Figure S6B,C) APOE4 mice displayed a rightward-shifted cumulative probability in the day 7 group compared to the day 1 group, while no significant difference was detected in the sham groups of APOE4 mice, indicating hyperexcitability on post-ISO day 7. Together, these findings indicate that a single 2 h ISO exposure produces consistently depressing effects on OB mitral cell excitability in APOE4 mice on both acute (day 1) and chronic (day 7) time scales compared to sham groups or compared to ISO-treated APOE3 mice on post-ISO day 1.

### 3.3. APOE4 Exacerbates ISO-Induced Neuronal Network Dysfunction in the OB of Awake Mice

We next analyzed OB LFP data to determine whether ISO altered network oscillations. On post-ISO day 1, APOE4 mice showed reduced PSD at 0–6 Hz (*p* < 0.05, Figure 3 inset a), indicating ISO-altered network operation. Interestingly, sham APOE4 mice showed stronger oscillatory power in the frequency range 0–49 Hz compared to the APOE3 sham group (Figure 3, insets b–d), suggesting APOE4-derived network dysfunction at the baseline level. However, no statistically significant differences were detected between ISO and sham groups of APOE4 mice on post-ISO day 7, between ISO and sham groups of APOE3 mice on post-ISO day 1 or day 7, or between APOE4 and APOE3 ISO groups on post-ISO day 1 or day 7. When comparing the post-ISO day 7 groups to day 1 groups, only a general reduction in the whole frequency range 0.1–100 Hz in APOE4 sham control groups (Figure S7) at the animal level was detected. The lack of statistical significance of these comparisons at the animal level may be due to the small-sized samples. Thus, these findings are just preliminary.

Together, these findings indicate that a single 2 h ISO exposure produces genotype-dependent alterations in OB network operation, which may contribute to olfactory behavioral deficits.

### 3.4. ISO Alters Synaptic Transmission in the OB and HI in APOE4 Mice

Given that ISO can modulate synaptic transmission [44], and that we observed lipid and OB network alterations after ISO exposure, we tested whether ISO altered excitatory and inhibitory inputs onto OB external tufted cells and hippocampal CA1 pyramidal neurons on day 7 after exposure (Figure 4 and Figure 5; Supplementary Figures S8 and S9). Unlike the in vivo recording cohorts, both Sham and ISO mice in the patch-clamp experiments underwent a brief laparotomy to control for surgical stress; Sham mice recovered shortly after surgery, whereas ISO/OP mice remained under ISO for a total exposure of 2 h. Whole-cell recordings measured spontaneous excitatory and inhibitory postsynaptic currents at −60 mV and 0 mV, respectively, with DNQX and gabazine confirming AMPA- and GABA_A_-mediated currents. ETC membrane resistance was lower in APOE4 Sham/OP mice than in APOE3 Sham/OP or APOE4/ISO/OP mice in the OB, whereas no corresponding differences were detected in HI PNs (Supplementary Figures S8 and S9).

At the synaptic level in the OB (Figure 4), spontaneous excitatory and inhibitory postsynaptic currents were recorded from ETCs and PNs voltage-clamped at −60 mV or 0 mV, respectively (Figure 4A). Complete blockades by DNQX or gabazine confirmed that sEPSCs and sIPSCs were mediated by AMPA and GABA_A_ receptors, respectively. In OB ETCs, APOE4/Sham mice showed significantly increased sEPSC frequency and amplitude compared with APOE3 Sham mice (Figure 4B,D), suggesting enhanced synaptic excitation. ISO/OP exposure reduced sEPSC frequency in both APOE3 and APOE4 mice, with modest increases in sEPSC amplitude, indicating an overall reduction in synaptic excitation. Similar to sham mice, APOE4/ISO/OP mice showed higher sEPSC frequency and amplitude than APOE3/ISO/OP mice, consistent with elevated synaptic excitation in APOE4 mice. In contrast, APOE4/Sham mice showed significantly reduced sIPSC frequency and amplitude compared with APOE3/Sham mice (Figure 4E–H), suggesting reduced synaptic inhibition. ISO exposure reduced both sIPSC frequency and amplitude in APOE3 mice, whereas in APOE4 mice, ISO reduced sIPSC frequency but increased sIPSC amplitude, suggesting opposing effects on inhibitory input strength and event frequency in OB ETCs.

Hippocampal CA1 PNs showed a distinct pattern (Figure 5). APOE4/Sham mice had significantly reduced sEPSC amplitude and frequency compared with APOE3/Sham mice (Figure 5A–D), indicating reduced hippocampal synaptic excitation. ISO/OP exposure increased sEPSC amplitude in APOE3 mice but reduced it in APOE4 mice, indicating opposing genotype-dependent effects. Similar to OB ETCs, ISO reduced sEPSC frequency in hippocampal PNs of both APOE3 and APOE4 mice. Among ISO/OP-exposed groups, APOE4 mice showed lower sEPSC amplitude and frequency than APOE3 mice, consistent with reduced synaptic excitation. For inhibitory transmission, APOE4 sham mice showed increased sIPSC amplitude and frequency compared with APOE3 sham mice (Figure 5E–H), opposite to the pattern observed in OB ETCs. ISO increased both sIPSC frequency and amplitude in APOE3 mice, whereas in APOE4 mice it increased sIPSC amplitude but reduced sIPSC frequency. Among ISO-exposed groups, APOE4 mice showed higher sIPSC amplitude but lower sIPSC frequency than APOE3 mice, again differing from the OB ETC pattern.

In summary, APOE4 increased synaptic excitation and reduced inhibition in OB ETCs, shifting the balance toward hyperexcitation. In contrast, ISO reduced both excitation and inhibition in APOE3 mice and produced mixed effects in APOE4 mice. In hippocampal CA1 PNs, APOE4 had the opposite baseline effect, reducing excitation and increasing inhibition. ISO shifted hippocampal synaptic balance toward inhibition in both APOE genotypes, while affecting excitation differently: increasing sEPSC amplitude in APOE3 mice but reducing it in APOE4 mice.

### 3.5. APOE4/ISO/OP Mice Show Persistent Olfactory and Neurological Dysfunction Linked to Chronic Transcriptomic Dysregulation

Finally, we assessed the long-term neurological effects of ISO/OP in asymptomatic young adult mice. Three-month-old male APOE3 and APOE4 mice underwent 2 h ISO/OP or sham treatment, and behavioral outcomes were measured longitudinally for up to 12 weeks after the intervention. Olfactory memory was assessed using the OM test, in which reduced exploration of a previously encountered odor indicates recognition. APOE4/ISO/OP mice showed persistent olfactory memory impairment beginning on day 11 and lasting for several weeks after ISO/OP (Figure 6A). Buried food testing further revealed transient impairment in odor-guided food localization in APOE4/ISO/OP mice compared with sham controls and APOE3/ISO/OP mice on days 12 and 37 (Figure 6B). Three-way repeated-measures ANOVA identified significant genotype × treatment interactions for both OM (*p* = 0.0068) and buried food performance (*p* = 0.0497), indicating differential responses to ISO/OP between APOE3 and APOE4 mice. Rotarod testing revealed early motor deficits in APOE4/ISO/OP mice during the first two weeks, which resolved thereafter (Figure 6C), while nest-building, grip strength, and open-field testing showed no significant group differences (Supplementary Figures S10 and S11). Cognitive testing revealed no early Y-maze differences, whereas APOE4/ISO/OP mice exhibited reduced spontaneous alternation and increased arm returns on day 86, without changes in total arm entries (Figure 6D–F). Although the overall genotype × treatment interactions were not significant for rotarod or Y-maze outcomes, the longitudinal analysis also identified genotype-dependent changes over time in Y-maze performance. NOR performance did not differ consistently among groups, although transient impairment was detected during the choice phase on day 41 (Supplementary Figures S10 and S12). Together, these findings demonstrate that ISO/OP exposure is associated with the earlier emergence of olfactory and motor abnormalities in APOE4 mice relative to age-matched sham controls, followed by delayed spatial working-memory impairment.

Having characterized early glial lipid alterations at day 7, we profiled OB transcriptomic changes at day 90 to examine the chronic transcriptional state at the conclusion of longitudinal behavioural testing. RNA-seq showed clear clustering by genotype and treatment (Figure 7A; *n* = 4–6 mice/group). Differential expression analysis with limma and FDR correction identified distinct differences between APOE4/ISO/OP and APOE3/ISO/OP mice (Figure 7B). GO enrichment showed upregulation of pathways related to membrane potential regulation and calcium ion transport (Figure 7C), and downregulation of metabolic, energetic, and protein-complex pathways (Figure 7D). Representative genes from these pathways are shown in Figure 7E,F. Lipid metabolism-related genes were also broadly reduced in APOE4/ISO/OP mice, including chaperones (*Hspa8*, *Hsp90b1*, and *Hsp90aa1*), stress-response genes (*Fos*, *Atf4*, and *Xbp1*), and lipid/bioenergetic genes (*Cyp51*, *Hmgcs1*, and *Msmol*; Figure 7G,H). However, these ISO/OP-associated changes were modest relative to baseline APOE3 and APOE4 genotype differences, which showed stronger transcriptomic separation and lipid metabolism pathway differences (Supplementary Figure S13). In contrast, APOE4/ISO/OP mice showed few OB DEGs compared with APOE4/Sham controls, whereas APOE3 mice exhibited more pronounced ISO-associated changes (Supplementary Figure S14A–J). Lipid metabolism analysis identified 11 DEGs in the APOE3/ISO/OP vs. APOE3/Sham comparison (Supplementary Figure S14K).

In the HI (Supplementary Figure S15), normalized counts also clustered by genotype and treatment, and APOE4/ISO/OP differed from APOE3/ISO/OP mice at FDR < 0.05. Enrichment analysis showed upregulation of RNA splicing pathways and downregulation of organelle and ribosome assembly pathways in APOE4/ISO/OP compared with APOE3/ISO/OP mice. Baseline genotype differences in the HI were more robust than ISO effects, with APOE4/Sham vs. APOE3/Sham showing enrichment for circadian rhythm and chromatin remodeling among upregulated genes, and muscle contraction and NADH regeneration among downregulated genes (Supplementary Figure S16). No DEGs were detected in APOE4/ISO/OP vs. APOE4/Sham, whereas APOE3/ISO/OP vs. APOE3/Sham showed more DEGs, mainly involving downregulated adherent’s junction assembly and apical protein localization pathways, with *Actb* and *Ccn3* as representative genes. Lipid metabolism-focused analysis identified *Ubc* as the only related DEG in both APOE4/Sham vs. APOE3/Sham and APOE3/ISO/OP vs. APOE3/Sham comparisons. Taken together, we found that ISO/OP induced long-lasting olfactory and late-emerging cognitive impairments specifically in APOE4 mice, alongside transcriptomic signatures in OB and HI that reveal disrupted metabolic, splicing, and ribosomal pathways.

To further determine whether APOE genotype modifies long-term transcriptional responses to ISO/OP, we performed a two-factor differential expression analysis incorporating genotype, treatment, and their interaction (full interaction results in Supplementary Materials 4). At 90 days after ISO/OP, the OB exhibited significant genotype × treatment interactions across multiple genes (FDR < 0.05), including *Nr4a3*, *Fosl2*, *Midn*, and *Hdac5* (Supplementary Figure S17A). In contrast, although several genes in HI showed nominal interaction effects (*p* < 0.05), none survived FDR correction (Supplementary Figure S17B). These findings demonstrate that APOE genotype significantly modifies persistent transcriptional responses to ISO/OP in a brain region-dependent manner, with stronger statistical evidence of genotype-dependent transcriptional responses in the OB than in the HI (Figure 8).

Schematic overview of the observation timeline following 2 h (h) isoflurane (ISO) anesthesia and laparotomy (ISO/OP) in young adult male humanized APOE4 and APOE3 mice. ISO/OP was followed by early olfactory bulb (OB) mitral cell layer circuit dysfunction at 1 day (d), altered OB circuit activity and glial lipid remodeling in the OB and hippocampus (HI) at 7 d, olfactory dysfunction at 11–12 d, and cognitive impairment at 86 d after surgery. Cell type-specific lipidomic profiling at 7 d revealed region- and cell type-dependent alterations in fatty acids, triglycerides, phospholipids, and sphingolipids in microglia and astrocytes. The arrows between illustrations link the components of the experimental overview, and the horizontal arrow below indicates the progression of time after ISO/OP exposure. Together, these findings identify APOE4-associated vulnerability to ISO/OP, marked by glial lipid dysregulation, olfactory circuit dysfunction, olfactory impairment, and delayed cognitive deficits.

## 4. Discussion

By examining how short-term ISO/OP exposure (Figure 8) interacts with the AD genetic risk factor APOE4, we identified APOE4-specific vulnerabilities in lipid metabolism, neural circuit function, and behavior of male mice. ISO/OP induced glial lipid dysregulation in the OB and HI, altered neural circuit function, and caused persistent olfactory deficits that preceded later cognitive dysfunction. Ninety days after ISO/OP exposure, transcriptomic analysis revealed alterations of calcium transport and metabolic pathways in APOE4 male mice, suggesting persistent genotype-associated molecular differences following ISO/OP. Collectively, these findings indicate that ISO/OP exacerbates APOE4-associated neurobiological changes involving glial lipid homeostasis, thereby impairing neural circuit integrity and contributing to progressive functional deficits.

Although surgical inflammation is a major contributor to postoperative cognitive dysfunction, anesthesia alone can also affect neural and behavioral outcomes. In our prior work with aged mice, ISO alone produced modest, transient changes, whereas combined anesthesia and surgery caused robust, sustained olfactory and cognitive impairments [7], supporting a synergistic effect. Thus, the behavioral, lipidomic, and transcriptomic experiments in the present study were interpreted within the combined ISO/OP model, which better reflects the clinical perioperative setting. The electrophysiological experiments used modality-specific designs. The in vivo recording cohort tested the effects of 2 h ISO exposure after recovery from the cranial preparation required for awake head-fixed recordings, without laparotomy at the time of ISO exposure. In contrast, the patch-clamp cohort compared Sham/OP and ISO/OP mice that both underwent laparotomy, with the major experimental difference being brief versus prolonged ISO exposure. This design controlled for laparotomy while supporting prolonged ISO exposure as a contributor to the synaptic alterations observed 7 days later. Nevertheless, because behavioral, lipidomic, and transcriptomic endpoints did not include a dedicated ISO-alone APOE4 cohort, those findings should be interpreted as responses to the combined ISO/OP paradigm rather than effects attributable specifically to ISO. Although our transcriptomic data suggested immune pathway activation after ISO/OP, especially in aged animals [7,45], the current study did not measure circulating or protein-level inflammatory biomarkers. Future studies incorporating peripheral and central immune readouts will help clarify the roles of inflammatory signaling and direct anesthetic effects.

Lipids, essential nutrients for all living organisms, play a critical physiological role in the brain, the second most lipid-rich organ after the adipose tissue, where they account for 10–12% of the brain’s fresh weight and over 50% of its dry weight [46]. In AD, aggregation of lipid droplets, dysfunctional lipid metabolism, and increased levels of lipid oxidation have been recognized as an important hallmark of neuropathology alongside amyloid plaques and neurofibrillary tangles [47,48]. In APOE4 carriers, lipid signatures of both neurons and glial cells include increased levels of Cer and FA [49,50,51], alterations that contribute to increased susceptibility to AD. Our findings suggest that the same underlying vulnerability may also be engaged by perioperative stress, as ISO/OP produced pronounced glial lipid dysregulation together with contemporaneous circuit abnormalities and subsequent behavioral deficits in APOE4 male mice. Although the present study does not establish direct causal mediation, these convergent findings support lipid dysregulation as a plausible contributor to APOE4-associated susceptibility to PND. Both OB- and HI-derived glial lipidomic profiling revealed that, even in young asymptomatic mice, short-term exposure to ISO/OP unmasked APOE4-specific functional deficits and lipid perturbations. Despite multiple prior studies reporting the effects of isoflurane and other anesthetics on lipid composition and peroxidation [52,53,54], our study provides direct evidence that APOE genotype modifies lipid responses to isoflurane anesthesia and surgery in the brain.

The lipidomic data supports a model in which ISO/OP induces cell type- and region-specific disruption of glial lipid homeostasis, with APOE4-associated changes most evident as broad remodeling of neutral lipids, phospholipids, lysophospholipids, sphingomyelins, ceramides, and hexosylceramides rather than as a single mitochondrial lipid signature. Our lipidomic analysis revealed distinct profiles between APOE4/ISO/OP and APOE3/ISO/OP glial cells across the OB and HI, characterized by alterations in membrane-associated lipids and selected TG and FA species. Given that TG stored in lipid droplets can sequester FAs and protect cells against lipotoxicity, the reduced TG species observed in APOE4/ISO/OP OB microglia relative to APOE3/ISO/OP microglia may reflect altered neutral lipid storage or diminished FA-buffering capacity [55,56]. This interpretation is consistent with prior evidence that APOE4 perturbs astrocytic fatty acid metabolism, lipid droplet formation, and fatty acid handling between neurons and astrocytes [30,31]. In the APOE4/ISO/OP versus APOE4/Sham comparison, however, the data more strongly support broad lipid remodeling after ISO/OP than a simple TG-depletion model, because several FA, TG, LPC/LPE, SM, PE/PC, HexCer, and Cer species shifted in a region- and cell type-dependent manner. Changes in sphingolipids and glycerophospholipids are biologically relevant because these lipid classes contribute to membrane structure, membrane fluidity, cell signaling, and neuroinflammatory regulation [57,58]. Together, these findings indicate that ISO/OP is associated with broad remodeling of glial lipid storage, fatty acid handling, and membrane lipid composition in APOE4 mice, consistent with disrupted lipid homeostasis rather than a single lipid class-specific mechanism.

Our lipidomic and physiological findings support the notion that OD may signal anesthesia- and APOE4-related vulnerability, appearing before overt cognitive decline. The OB, being highly metabolically active and among the earliest brain regions to exhibit APOE4-driven pathology [59], is particularly susceptible to additional metabolic stressors such as general anesthesia and surgery. We observed lipid dysregulation in the OB, including TG depletion, phospholipid remodeling, and accumulation of bioactive sphingolipids, suggesting impaired lipid storage and trafficking capacity in glial cells, which normally buffer neurons against energetic fluctuations. Isoflurane’s effects on neuronal excitability and synaptic balance were strongly modulated by APOE genotype, potentially related to lipidomic dysregulation in astrocytes and microglia that are critical regulators of brain homeostasis [60,61]. Astrocytes, the brain’s primary source of APOE, are responsible for cholesterol and phospholipid transport to neurons [60,62]. In APOE4 male mice, deficits in astrocytic lipid handling impair membrane maintenance, and exposure to isoflurane likely worsens these disruptions, altering neuronal and glial membrane composition and affecting synaptic function. Microglia, key players in synaptic pruning and inflammation, are also sensitive to lipid imbalance. Isoflurane-induced changes in lipids like ceramides and sphingolipids can activate microglia, particularly in APOE4 male mice where lipid turnover is already impaired [63]. This may underline the observed mitral cell hypoexcitability and altered theta oscillations in APOE4 male mice. Conversely, APOE3 astrocytes and microglia may more effectively restore lipid homeostasis following isoflurane exposure, leading to transient hyperexcitability without long-term circuit dysfunction. Collectively, these findings suggest that OD reflects not only local circuit vulnerability but also serves as an early, functionally accessible readout of APOE4- and anesthesia-associated disruptions in lipid metabolism and neuronal–glial energetics.

The electrophysiological experiments used two related but distinct cohort designs, both involving surgical preparation but differing in the timing and type of procedure relative to ISO exposure. In vivo OB recordings tested the effect of a 2 h ISO exposure after recovery from the cranial preparation required for awake head-fixed recording. By contrast, the patch-clamp cohort compared Sham/OP and ISO/OP mice that both underwent laparotomy, with the principal difference being brief versus prolonged ISO exposure. Thus, the patch-clamp recordings served as a follow-up experiment to further characterize ISO-associated electrophysiological alterations observed in vivo while controlling for laparotomy in the ex vivo synaptic-transmission experiments. This timing supports the conclusion that the electrophysiological findings reflect post-exposure circuit or synaptic alterations rather than residual anesthetic effects, consistent with prior evidence that brain ISO concentrations fall below detectable levels within minutes after discontinuation in mice [64]. Thus, although ISO concentrations were not directly measured in our own animals, the day 1 findings and their differences from those observed on day 7 are best viewed as early post-exposure circuit alterations rather than as direct effects of residual anesthetic.

Building on Foley et al.’s report of distinct APOE4-driven cortical gene signatures in young mice [65], our findings provide further evidence that APOE genotype influences long-term neurological and molecular responses to ISO/OP. Three-way ANOVA of longitudinal behavioral outcomes identified significant genotype × treatment interactions in odor memory and buried food performance, supporting differential olfactory responses to ISO/OP between APOE3 and APOE4 mice. At the transcriptional level, formal interaction analysis identified multiple genes exhibiting significant genotype × treatment interactions in the OB at 90 days after ISO/OP, whereas no significant interactions survived FDR correction in the HI. These findings indicate that APOE genotype modifies persistent transcriptional responses to ISO/OP, particularly in the OB. Interestingly, despite pronounced neurological impairments, APOE4 mice exhibited relatively few treatment-associated DEGs compared with APOE3 mice. This muted transcriptional response may reflect an altered or pre-stressed molecular state [66,67,68], in which baseline changes in metabolism, stress responses, and cellular homeostasis limit further transcriptional adaptation. Importantly, previous studies have demonstrated age-dependent neurological abnormalities in humanized APOE4 knock-in mice, including olfactory dysfunction emerging by 6–8 months of age [69]. As animals in the present study approached this age at the later assessment points, spontaneous progression of APOE4-associated abnormalities in sham controls may have reduced the detectable differences between ISO/OP and Sham groups. Thus, relatively modest treatment-associated differences at later endpoints do not necessarily indicate an absence of earlier exposure-induced perturbations or their subsequent resolution. Together, these findings suggest that APOE4-associated vulnerability to perioperative stress involves both altered olfactory function and region-specific transcriptional responses, although the relationship between these changes remains to be established.

A previous study showed that anesthesia/surgery induces olfactory and cognitive impairment and proposed an IL-6-mediated pathway linking olfactory dysfunction to hippocampal changes [4]. Our study extends these findings by examining APOE4-associated vulnerability to perioperative stress, particularly at the level of glial lipid metabolism and olfactory circuit function. The additional longitudinal statistical analysis further supports genotype-dependent vulnerability to perioperative olfactory dysfunction. Three-way ANOVA identified significant genotype × treatment interactions for both odor memory and buried food performance, demonstrating that the effects of ISO/OP on olfactory outcomes differed between APOE3 and APOE4 mice. In contrast, genotype × treatment interactions were not significant for rotarod or Y-maze outcomes, despite the observed time-specific impairments in APOE4/ISO/OP mice. Together, these findings support APOE4-associated susceptibility to postoperative olfactory dysfunction while highlighting the distinct temporal patterns of neurological impairment, with olfactory abnormalities emerging before detectable spatial working-memory deficits.

The different trajectories of buried food and odor memory performance may reflect their distinct processing demands. Buried food testing assesses odor-guided food localization, whereas odor memory testing additionally assesses retention and recognition of a previously encountered odor [70,71]. Recovery of food-finding performance may therefore coexist with persistent impairment in recognition-related processing, without implying complete recovery of olfactory sensitivity. The specific neural mechanisms underlying these different trajectories remain to be determined. Memory impairments also differed across tasks: APOE4/ISO/OP mice showed impaired odor memory over a 1 h retention interval and delayed impairment in spatial working memory, whereas the 24 h NOR test did not reveal a consistent ISO/OP-associated deficit. These findings suggest predominant impairment of short-term odor memory and spatial working memory within the observation period, although differences in sensory modality and task demands may also contribute. The absence of a consistent NOR deficit does not exclude underlying APOE4-related vulnerability or subsequent deterioration in object recognition memory.

As with other humanized APOE knock-in studies, species-specific differences and the use of 100% oxygen during anesthesia should be considered when extrapolating these findings to clinical populations and interpreting the lipidomic findings. Supplemental oxygen is commonly used in mouse anesthesia to minimize hypoxemia, and Wilding et al. found that 100% versus 21% oxygen did not alter major physiological variables in isoflurane-anaesthetized mice, although continuous nose-cone administration of 100% oxygen increased atelectasis [72]. Importantly, short-term normobaric hyperoxia does not invariably produce cerebral oxidative injury. In patients with severe traumatic brain injury, exposure to an FiO_2_ of 1.0 for 2 h markedly increased arterial and brain tissue oxygenation without increasing CSF F_2_-isoprostanes or protein oxidation or altering antioxidant measures [73]. Similarly, in mice, 2 h of 100% oxygen alone did not increase cerebral nitrotyrosine in sham animals, although hyperoxia augmented oxidative injury when administered after hypoxic–ischemic injury, suggesting that its redox consequences depend on the underlying physiological state [74]. Avoidance of hypoxemia may be particularly relevant to APOE4, as mice expressing human APOE4 exhibit increased susceptibility to intermittent hypoxia [75], while direct comparison of APOE3- and APOE4-targeted replacement mice demonstrated impaired cerebral perfusion and greater hypoxia-associated white-matter injury in APOE4 mice during cerebral hypoperfusion [76]. Thus, reducing inspired oxygen would not necessarily constitute a biologically neutral alternative in this model. Nevertheless, because oxygen concentration was not independently manipulated in the present study, a contribution of elevated inspired oxygen to the observed lipid responses cannot be excluded. These findings should therefore be interpreted as responses to the combined ISO/OP paradigm rather than effects attributable specifically to isoflurane.

Another limitation is the use of male-only cohorts. Although sex modifies APOE4-associated phenotypes, APOE4-related effects are observed in both sexes, with sex often influencing the magnitude or trajectory of these effects rather than their presence [77,78,79,80]. Male mice were selected as an initial cohort to determine whether APOE4 confers susceptibility to perioperative glial lipid dysregulation and neurobehavioral deficits relative to APOE3. Direct comparison of male and female responses to perioperative ISO exposure remains an important next step, particularly given evidence that APOE4-related metabolic and cognitive phenotypes may be more pronounced in females.

Lastly, the small number of animals per group in both in vivo and in vitro electrophysiology experiments is an explicit limitation of this study, and larger cohorts will be needed to corroborate the preliminary findings.

From a translational standpoint, these findings support OD assessment, network dysfunction, and lipidomic profiling as potential early markers of perioperative vulnerability, particularly in APOE4 carriers. Routine olfactory testing and circulating lipidomic signatures, including extracellular vesicle-derived lipids, may help identify patients at higher risk for PND or accelerated neurodegeneration and inform clinical trial stratification or personalized perioperative monitoring. Because this study focused on isoflurane, future work should determine whether these lipid and circuit disruptions extend to other anesthetic agents. Interventions that stabilize lipid homeostasis or support mitochondrial function may also offer strategies to mitigate perioperative risk in genetically vulnerable patients.

In summary, our multidisciplinary approach demonstrates that APOE4 male mice are particularly vulnerable to perioperative stress, with ISO/OP associated with glial lipid dysregulation, circuit instability, and behavioral deficits. These alterations were evident at metabolic and circuit levels well before the broad transcriptional changes detected at day 90, supporting lipidomic changes and olfactory dysfunction as early indicators of vulnerability. By integrating glial lipid, circuit, behavioral, and transcriptomic findings across distinct time points, our study highlights APOE genotype as an important factor in perioperative neurological risk and provides a framework for future studies of anesthetic choice, patient stratification, and targeted intervention.

## Figures and Tables

**Figure 1:**
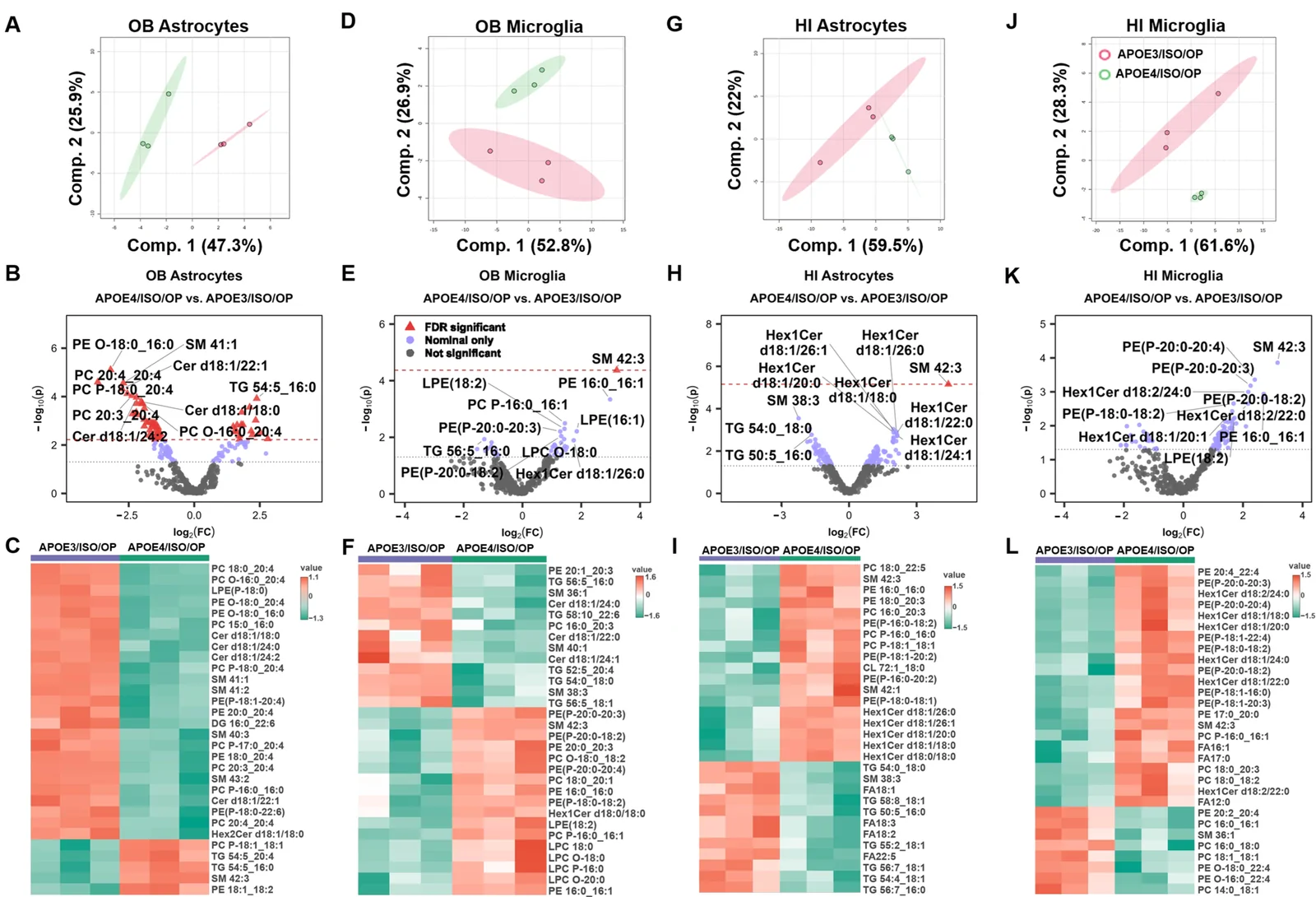
APOE4 drives heightened lipid dysregulation in olfactory bulb (OB) and hippocampal (HI) astrocytes and microglia 7 days after isoflurane (ISO) exposure and laparotomy operation (OP). (**A**–**C**) OB astrocytes, (**D**–**F**) OB microglia, (**G**–**I**) HI astrocytes, and (**J**–**L**) HI microglia from APOE4/ISO/OP vs. APOE3/ISO/OP mice at 7 days post-exposure. In each set, Partial least squares discriminant analysis (PLS-DA) illustrates sample clustering. Volcano plots display log_2_ fold changes and nominal *p*-values, with red triangles indicating lipids meeting the Benjamini–Hochberg false discovery rate (FDR)-adjusted significance threshold (adjusted *p* ≤ 0.05), purple points indicating nominal significance only (*p* < 0.05), and grey points indicating non-significant lipids. The red dotted horizontal line represents the nominal *p*-value cutoff corresponding to the FDR-adjusted significance threshold for each comparison. Heatmaps show the top 30 lipids selected by nominal Student’s *t*-test *p*-values for exploratory visualization. *n* = 3 pooled biological samples/group, each comprising three male mice (9 mice/group).

**Figure 2:**
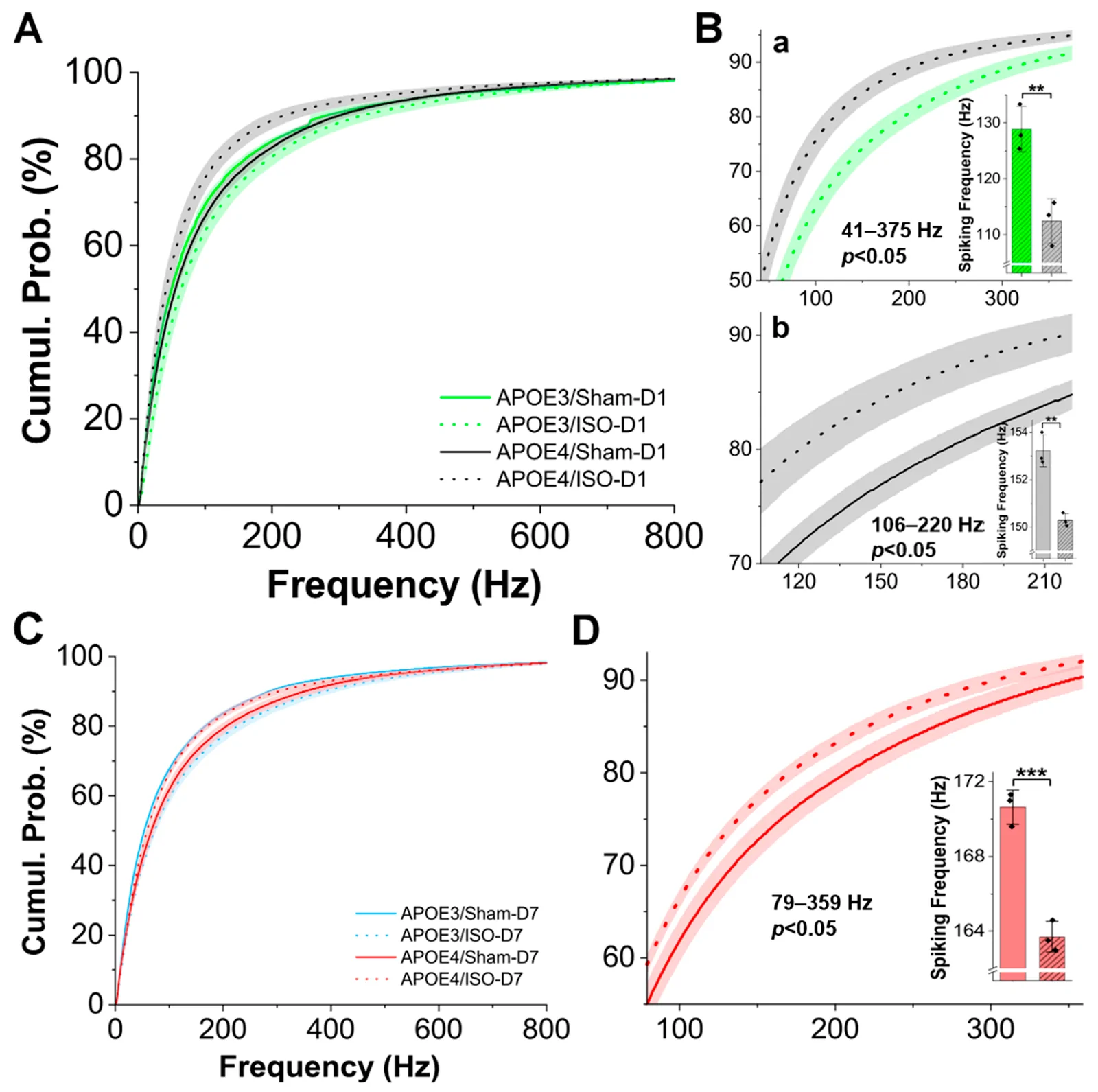
Isoflurane (ISO) effects on neuronal excitability in the OB mitral cell layer on post-ISO day 1 (D1) and 7 (D7). Line graphs were compared using the number of animals as the sample size and with the false discovery rate (FDR) correction to identify the frequency ranges with statistical significance. Bar graphs represent the averaged spike frequency within the corresponding frequency range with statistical significance from line graphs on the left and were analyzed using a two-sample *t*-test, with the animal as the experimental unit. (**A**) Comparison of the cumulative probability of spiking frequency on D1 post-ISO exposure among four groups of mice: (1) APOE3/sham (APOE3/Sham-D1, solid green curve, *n* = 3 mice with 7 recordings), (2) APOE3/ISO (APOE3/ISO-D1, dashed green curve, *n* = 3 mice with 6 recordings), (3) APOE4/sham (APOE4/Sham-D1, solid black curve, *n* = 3 mice with 6 recordings), (4) APOE4/ISO (APOE4/ISO-D1, dashed black curve, *n* = 3 mice with 6 recordings). (**B**) Enlarged view of A showing the specified frequency ranges with statistically significant differences between (**a**) APOE3/Sham-D1 and APOE3/ISO-D1groups and (**b**) APE4/Sham-D1 and APOE4/ISO-D1 groups at the animal level. (**C**) Comparison of the cumulative probability of spiking frequency on D7 post-ISO exposure among four groups of mice: (1) APOE3/sham (APOE3/Sham-D7, solid blue curve, *n* = 3 mice with 8 recordings), (2) APOE3/ISO (APOE3/ISO-D7, dashed blue curve, *n* = 3 mice with 5 recordings), (3) APOE4/sham (APOE4/Sham-D7, solid red curve, *n* = 3 mice with 6 recordings), and (4) APOE4/ISO (APOE4/ISO-D7, dashed red curve, *n* = 3 mice with 8 recordings). (**D**) Enlarged view of (**C**) showing the frequency range 79–359 Hz with statistically significant differences at the animal level between APOE4/Sham-D7 and APOE4/ISO-D7 groups. ** *p* < 0.01 and *** *p* < 0.001.

**Figure 3:**
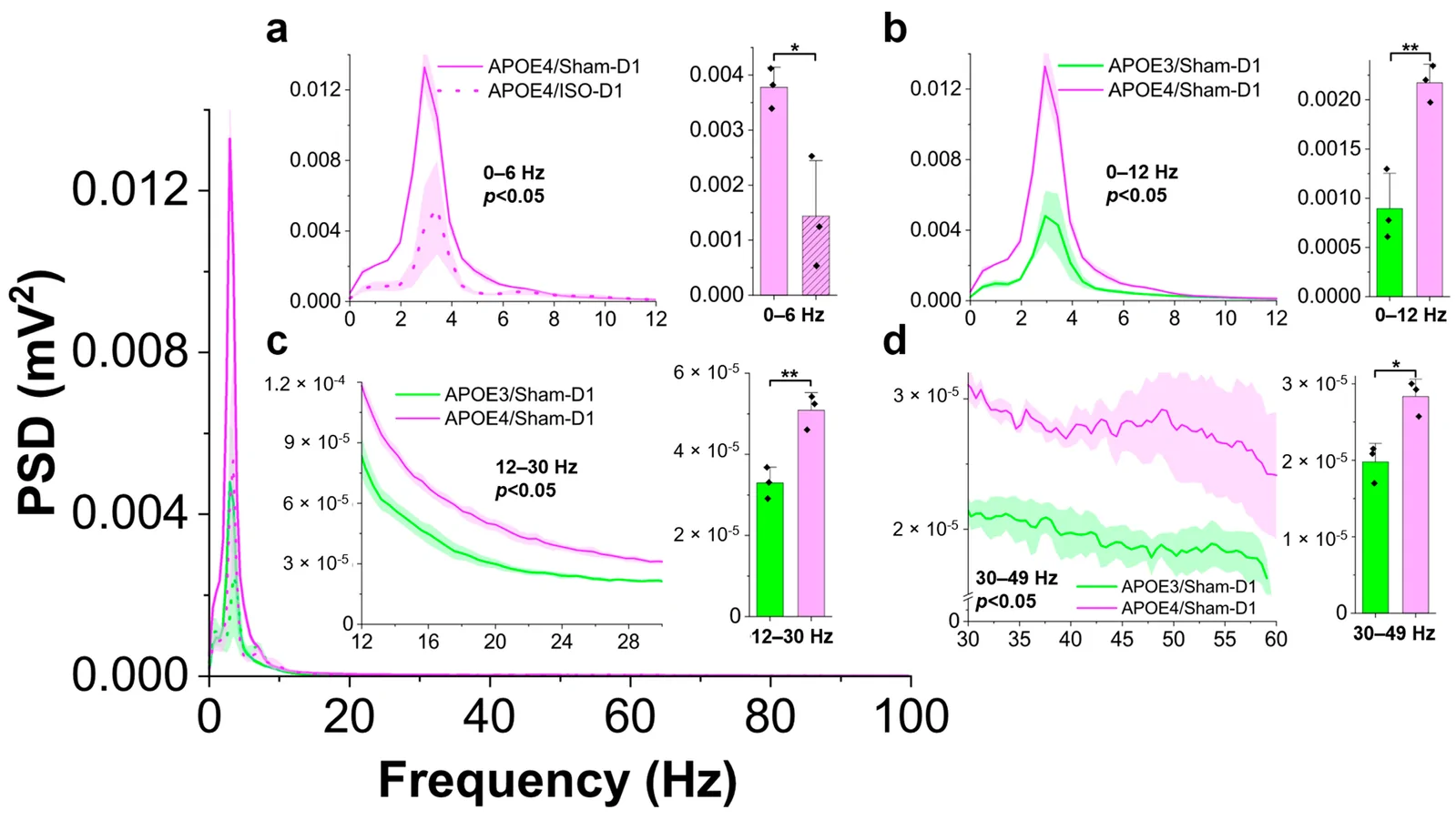
Isoflurane (ISO) effects on local field potential (LFP) oscillations of on post-treatment days 1 (D1) and 7 (D7). Line graphs were compared using the number of animals as the sample size, with the false discovery rate (FDR) correction applied, whereas the bar graphs represent the averaged power spectral density (PSD) within the corresponding frequency range with statistical significance on the left and were analyzed using a two-sample *t*-test with the animal as the experimental unit. The main panel shows the comparison of the PSD of LFP oscillatory activities on D1 post- ISO-exposure among four groups of mice (n-3 mice/group): (1) APOE3/Sham (APOE3/Sham-D1, solid green curve, 7 recordings), (2) APOE3/ISO (APOE3/ISO-D1, dashed green curve, 6 recordings), (3) APOE4/Sham (APOE4/Sham-D1, solid purple curve, 6 recordings), and (4) APOE4/ISO (APOE4/ISO-D1, dashed purple curve, 6 recordings). Inset (**a**) shows the comparison between APOE4 ISO and APOE4 sham groups on post- ISO-exposure day 1 (D1), with statistical significance in the frequency range 0–6 Hz. Insets (**b**–**d**) represent the comparison between APOE4 and APOE3 sham groups on day 1 in the frequency ranges 0–12 Hz (**b**), 12–30 Hz (**c**), and 30–49 Hz, which show statistical significance. Each curve (solid or dotted) with shaded area presents mean ± SE. * *p* < 0.05 and ** *p* < 0.01.

**Figure 4:**
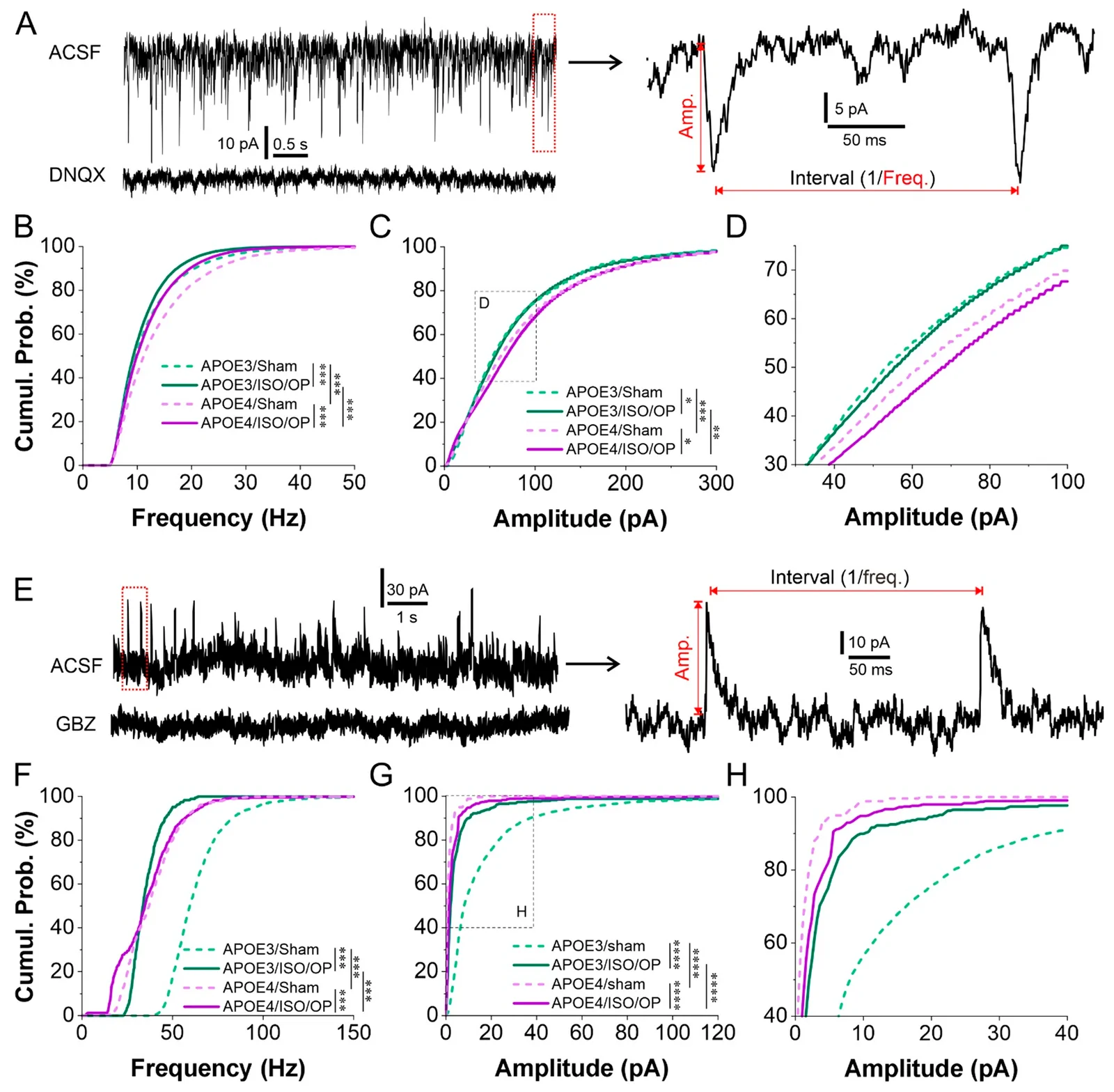
Effects on spontaneous excitatory (sEPSCs) and inhibitory (sIPSCs) postsynaptic currents in external tufted cells (ETCs) in the olfactory bulb (OB). (**A**) left: typical voltage clamp recording traces showing sEPSCs (top) that were eliminated by bath application of 10 mM DNQX, a selective AMPA receptor blocker (bottom); right: blown up from the left illustrating the measurement of sEPSC amplitude (amp.) and inter-EPSC interval. (**B**,**C**) Comparisons of sEPSC frequency (**B**) and amplitude (**C**) among four groups of mice: APOE3/Sham (*n* = 8 cells in 2 mice), APOE3/ISO/OP (*n* = 5 cells in 3 mice), APOE4/Sham (*n* = 9 cells in 3 mice), and APOE4/ISO/OP (*n* = 5 cells in 3 mice). (**D**) Blown up from the dashed box area in (**C**). (**E**) left: typical voltage clamp recording traces showing sIPSCs (top) that were eliminated by bath application of 10 mM gabazine (GBZ), a selective GABA_A_ receptor blocker (bottom); right: blown up from the left illustrating the measurement of sIPSC amplitude (amp.) and inter-IPSC interval. (**F**,**G**) Comparisons of sIPSC frequency (**F**) and amplitude (**G**) among four groups of mice: APOE3/Sham (*n* = 5 cells in 2 mice), APOE3/ISO/OP (*n* = 4 cells in 3 mice), APOE4/Sham (*n* = 5 cells in 3 mice), and APOE4/ISO/OP (*n* = 7 cells in 3 mice). (**H**) Blown up from the dashed box area in (**G**). Comparison between the two groups was analyzed using Kolmogorov–Smirnov (K–S) test. * *p* < 0.05, ** *p* < 0.01, *** *p* < 0.001, and **** *p* < 0.0001.

**Figure 5:**
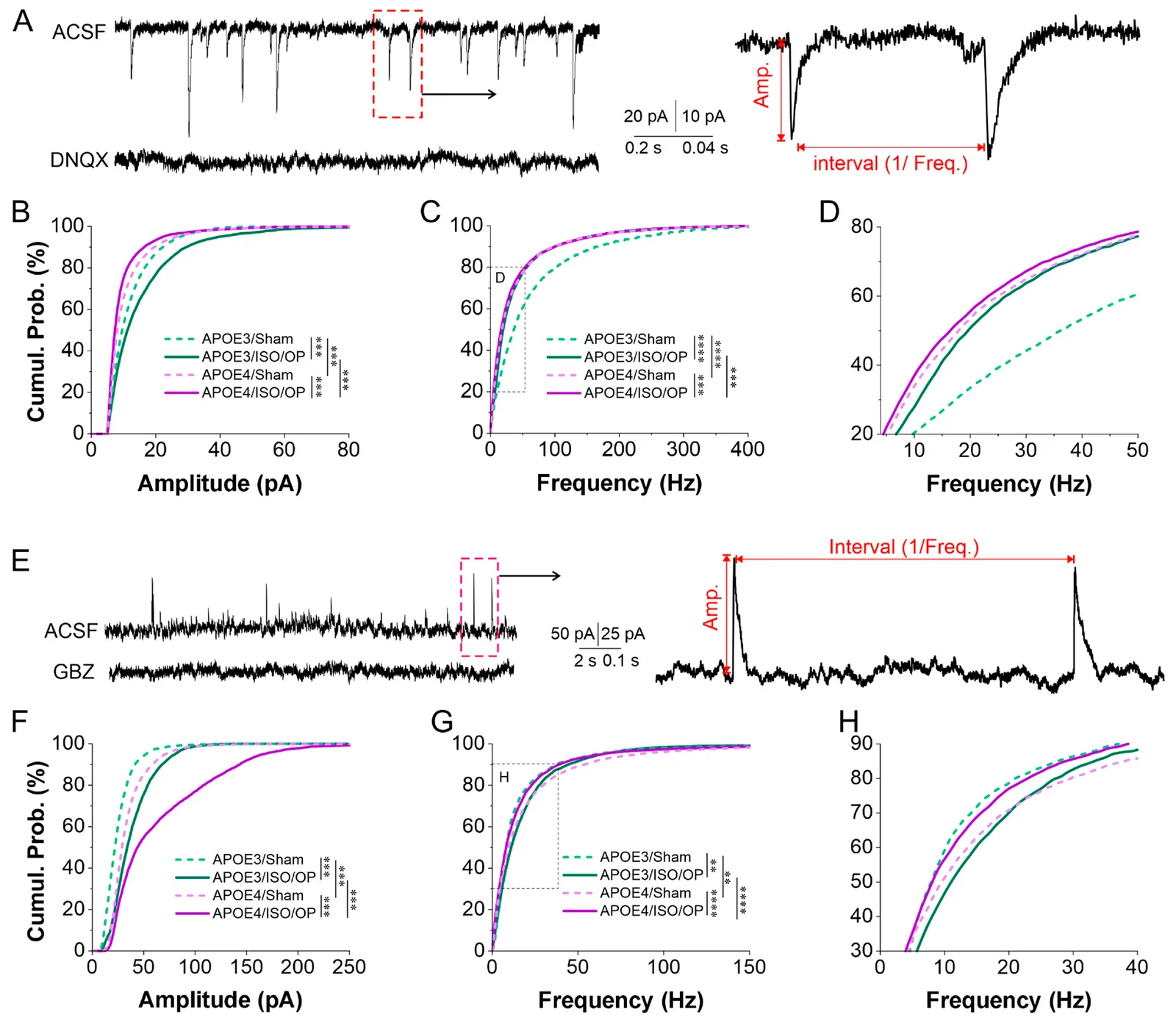
Effects on spontaneous excitatory (sEPSCs) and inhibitory (sIPSCs) postsynaptic currents in pyramidal neurons in the hippocampus (HI). (**A**) left: typical voltage-clamp recording traces showing sEPSCs (top) that were eliminated by bath application of 10 mM DNQX, a selective AMPA receptor blocker (bottom); right: blown up from the left illustrating the measurement of sEPSC amplitude (amp.) and inter-EPSC interval. (**B**,**C**) Comparisons of sEPSC amplitude (**B**) and frequency (**C**) among four groups of mice: APOE3/Sham (*n* = 8 cells in 2 mice), APOE3/ISO/OP (*n* = 9 cells in 3 mice), APOE4/Sham (*n* = 2 cells in 3 mice), and APOE4 ISO/OP (*n* = 15 cells in 3 mice). (**D**) Blown up from the dashed box area in C. (**E**) left: typical voltage clamp recording traces showing sIPSCs (top) that were eliminated by bath application of 10 mM gabazine (GBZ), a selective GABA_A_ receptor blocker (bottom); right: blown up from the left illustrating the measurement of sIPSC amplitude (amp.) and inter-IPSC interval. (**F**,**G**) Comparisons of sIPSC amplitude (**F**) and frequency (**G**) among four groups of mice: APOE3/Sham (*n* = 8 cells in 2 mice), APOE3/ISO/OP (*n* = 7 cells in 3 mice), APOE4/Sham (*n* = 12 cells in 3 mice), and APOE4/ISO/OP (*n* = 12 cells in 3 mice). (**H**) Blown up from the dashed box area in (**G**). Comparison between the two groups was analyzed using Kolmogorov–Smirnov (K–S) test., ** *p* < 0.01, *** *p* < 0.001, and **** *p* < 0.0001.

**Figure 6:**
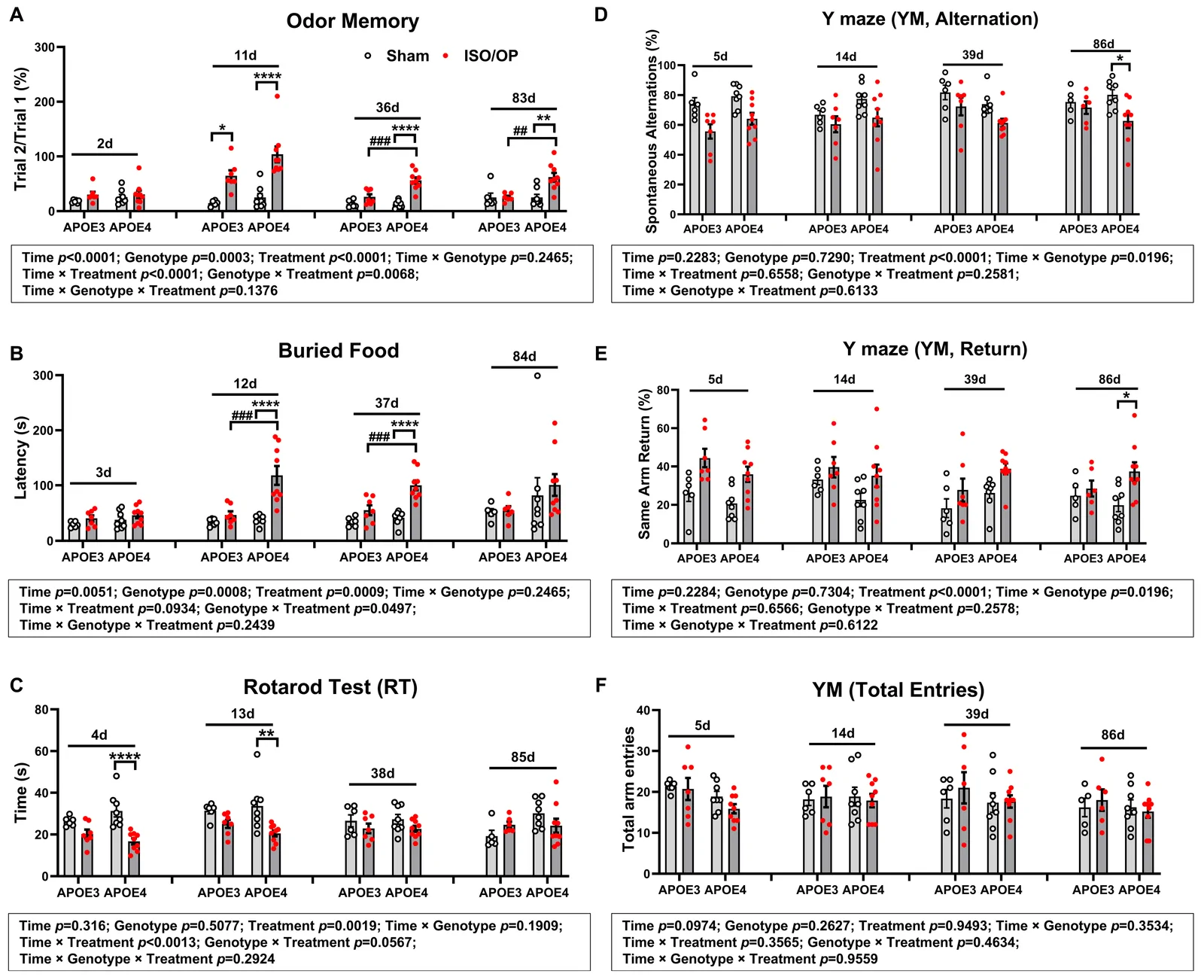
Isoflurane (ISO) exposure and surgery induce persistent olfactory dysfunction and delayed cognitive impairment in APOE4 mice. (**A**,**B**) Olfactory function was assessed using the odor memory (OM, (**A**)) and buried food (BF, (**B**)) tests. Increased Trial 2/Trial 1 sniffing ratios indicate impaired odor recognition memory, whereas increased latency to locate buried food indicates reduced olfactory sensitivity. (**C**) Motor coordination was evaluated using the rotarod (RT) test, with reduced latency to fall indicating impaired motor performance. (**D**–**F**) Spatial working memory was assessed using the Y-maze test, which revealed reduced spontaneous alternation (**D**) and increased same-arm returns (**E**) in APOE4/ISO/OP mice at day 86, without significant changes in total arm entries (**F**). Data are presented as mean ± SEM with individual animals shown. *n* = 6 (APOE3/Sham), 7 (APOE3/ISO/OP), 8 (APOE4/Sham), and 9 (APOE4/ISO/OP) mice. Pairwise comparisons at individual time points were performed using two-way ANOVA followed by Tukey’s multiple-comparisons test. Data were analyzed using three-way repeated-measures ANOVA to assess the main effects and interactions of time, genotype, and treatment. Main effects and interaction *p*-values are presented below each panel. * *p* < 0.05, ** *p* < 0.01, and **** *p* < 0.0001 versus genotype-matched Sham controls; ## *p* < 0.01 and ### *p* < 0.001 versus APOE3/ISO/OP at the corresponding time point.

**Figure 7:**
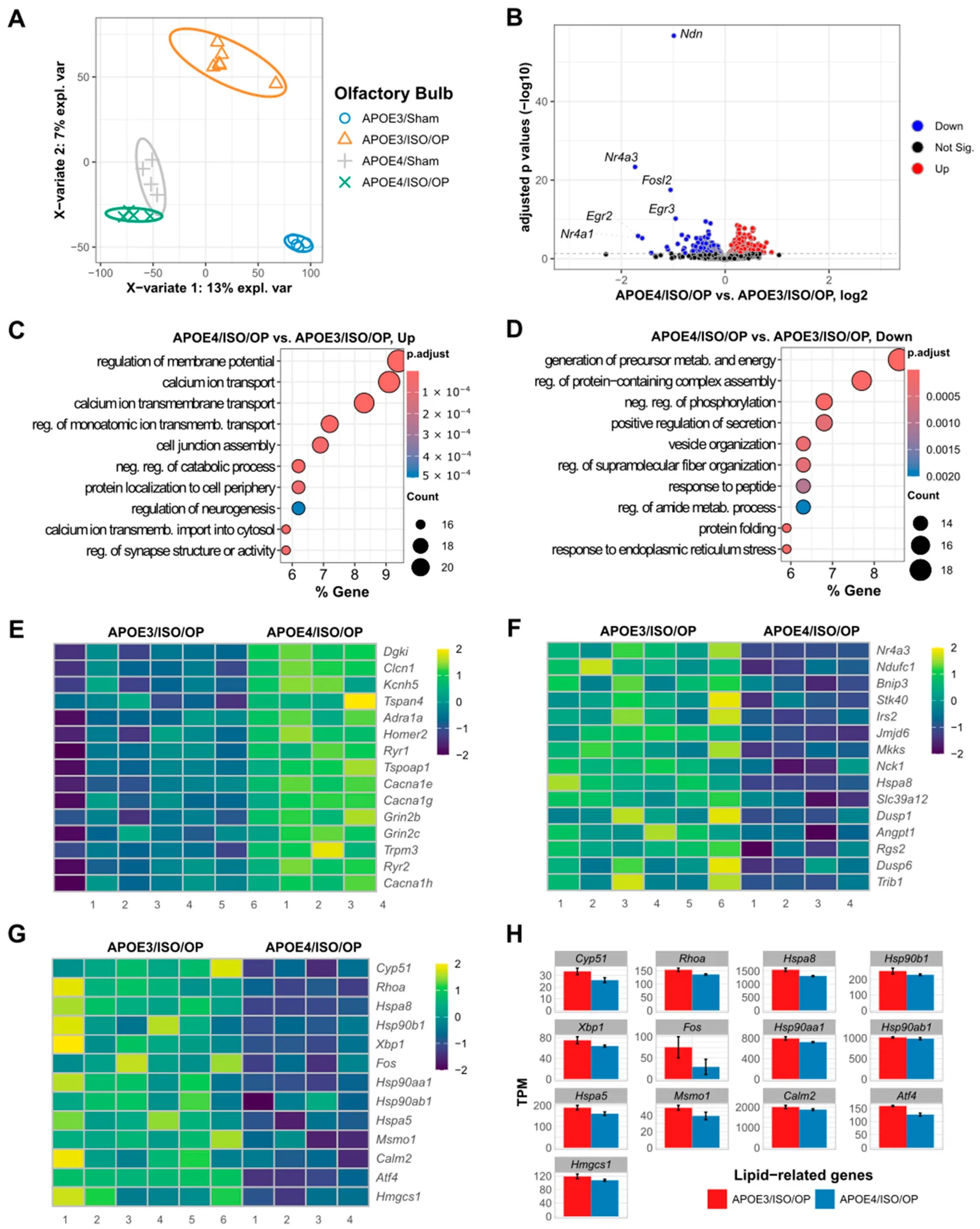
APOE4 is associated with altered olfactory bulb transcriptomic profiles 90 days after isoflurane exposure and laparotomy. (**A**) PLS-DA plot for all normalized transcriptome genes is depicted for humanized APOE4 and APOE3 mice with or without ISO exposure. (**B**) Volcano plot displaying differentially expressed genes (DEGs) in the APOE4/ISO/OP vs. APOE3/ISO/OP comparison. (**C**,**D**) Pathway enrichment analysis of upregulated (**C**) and downregulated (**D**) DEGs with Gene Ontology biological processes. (**E**,**F**) Heatmaps displaying DEGs associated with the top three upregulated pathways (**E**) and top three downregulated pathways (**F**) identified between APOE4/ISO/OP and APOE3/ISO/OP groups. Z-score normalization was applied to gene expression values. Heatmaps illustrate sample-wise variation (columns) across genes (rows). Color intensity reflects relative transcript abundance across samples: warmer colors for higher, cooler for lower. (**G**,**H**) Heatmap (**G**) and bar graph (**H**) showing lipid metabolism-related genes that were differentially expressed between APOE4/ISO/OP and APOE3/ISO/OP groups. All genes shown met the significance criterion of FDR < 0.05 after correction for multiple testing. *n* = 4–6 mice/group.

**Figure 8:**
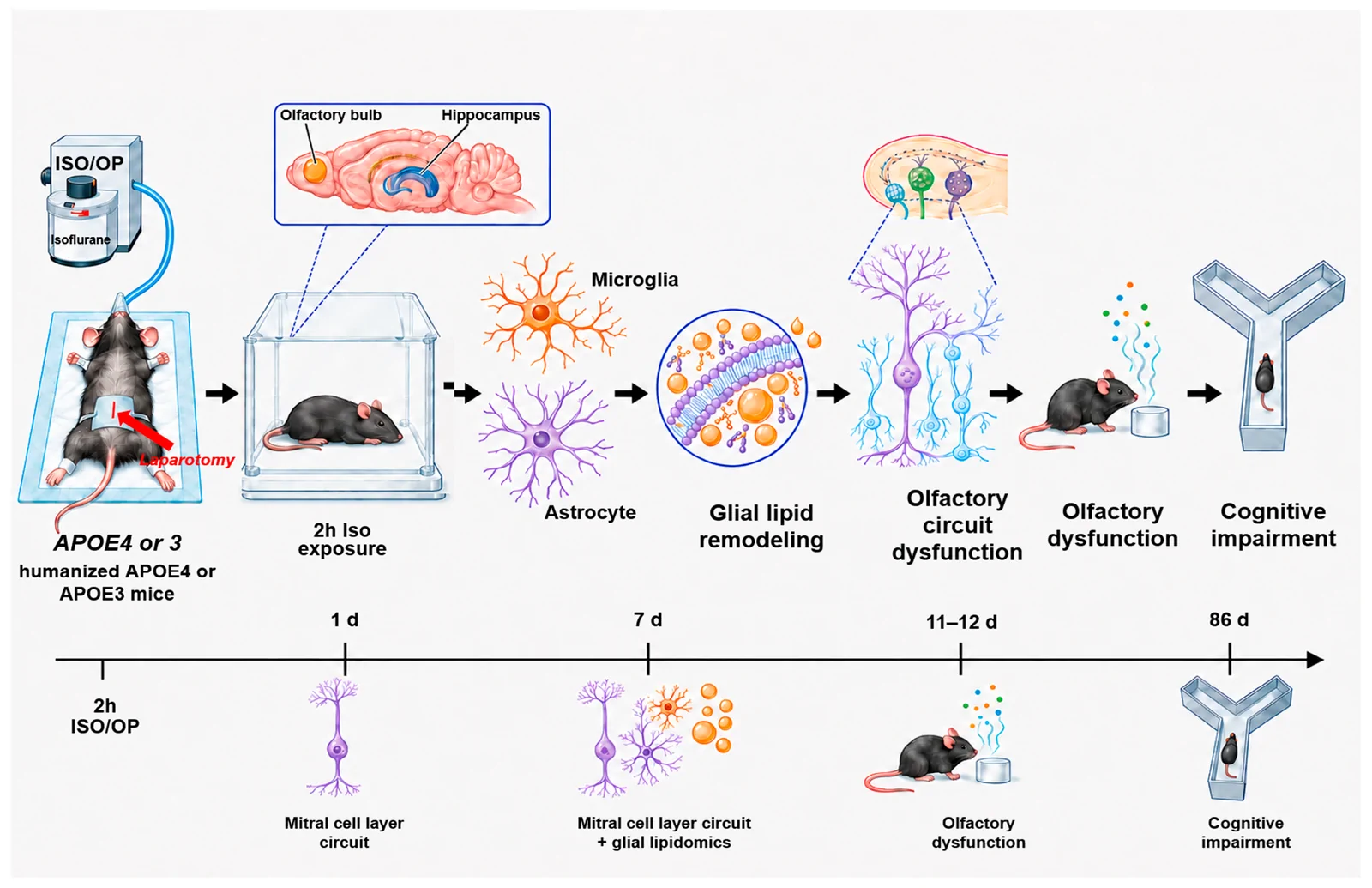
Experimental timeline of APOE4-dependent lipid dysregulation, neural dysfunction, and neurological impairment following isoflurane exposure and surgery in male mice.

## Data Availability

All data supporting the conclusions of this study are provided in the main text and/or in the Supplementary Materials, which include 17 Supplemental Figures with their corresponding legends.

## Supplementary Materials

The following supporting information can be downloaded at https://www.mdpi.com/article/10.3390/cells15212038/s1, Supplemental Materials 1: Extended Methods; Supplemental Materials 2: Additional figures; Supplemental Materials 3: Complete lipidomic differential abundance analysis and FDR; Supplemental Materials 4: RNAseq Interaction Results. References [81,82,83,84,85,86,87,88] are cited in the Supplementary Materials.
