# Supplementary material for: Isoflurane and Surgery Aggravate APOE4-Dependent Lipid Dysregulation, Neural Dysfunction, and Neurological Impairment in Male Mice
Source: Cells. 2026 Nov 9;15(21):2038. doi: 10.3390/cells15212038 (PMC13762270; doi:10.3390/cells15212038)
Supplement: Supplementary file 1 [file cells-15-02038-s001.zip › Supple Materials 1_Extended Methods 10-24-2026.pdf]

Supplemental Materials

Materials and Methods

Mouse general anesthesia and operation

Homozygous humanized APOE4 KI [B6(SJL)-Apoetm1.1(^{APOE*4})Adiuj/J, stock no. 027894] and APOE3 KI [B6.Cg-Apoeem2$^{(APOE*)}$Adiuj/J, stock no. 029018] mice (Mus musculus; male; 10-14 weeks old; 20-25 g) were obtained from The Jackson Laboratory. These knock-in mice carry targeted replacement of the endogenous mouse *Apoe* gene with human *APOE* sequences. Animals were maintained under specific pathogen-free conditions and housed under a 12-hour light/dark cycle with ad libitum access to food and water. GA was induced and maintained with 2% isoflurane (ISO) in 100% oxygen via a precision vaporizer. Following induction, animals were transferred to a surgical station and maintained under continuous ISO administration via a nose cone. During the surgical phase, anesthetic depth was assessed by monitoring respiratory rate and pattern, as well as palpebral and pedal reflexes. Core body temperature was maintained using a temperature-regulated heating pad. A midline laparotomy was performed through a 1.5 cm incision extending from the xiphoid process to 0.5 cm above the pubic symphysis, sequentially incising the skin, abdominal musculature, and peritoneum. At the conclusion of the procedure, the incision site was infiltrated with 0.25% bupivacaine in sterile saline and closed in layers using 5-0 monofilament nylon sutures. The surgical procedure lasted approximately 10 minutes. Following surgery, animals were returned to the anesthesia chamber and maintained under 2% ISO to complete a total exposure time of 2 h. During this period, respiratory rate and pattern were continuously monitored to ensure stable anesthetic depth, and no signs of respiratory distress or instability were observed. All animals were maintained under identical anesthetic conditions.

Postoperatively, mice were placed on a temperature-controlled pad for 30-60 minutes to facilitate thermal recovery. Animals were closely monitored for 4 h following general anesthesia and surgery, with continued daily assessments thereafter.

Adult cell isolation

Dissected OB and hippocampus (HI) were collected in ice-cold Hank's Balanced Salt Solution (HBSS) and enzymatically dissociated into single-cell suspensions using the Adult Brain Dissociation Kit (Cat# 130-107-677, Miltenyi Biotec), following the manufacturer's protocol. Homogenates were passed through a 70-μm cell strainer to remove debris and washed with HBSS supplemented with 0.5% bovine serum albumin (BSA). Myelin and cellular debris were eliminated by density gradient centrifugation with the Debris Removal Solution. The resulting suspension was resuspended in MACS buffer (PBS, 0.5% BSA, 2 mM EDTA) and incubated with

FcR Blocking Reagent to minimize nonspecific binding. For microglial isolation, cells were labeled with CD11b Microbeads (Cat# 130-093-634, Miltenyi Biotec), whereas astrocytes were enriched in parallel using ACSA-2 Microbeads (Cat# 130-097-678, Miltenyi Biotec). Magnetically labeled cells were applied to LS separation columns placed in a MACS Separator (Miltenyi Biotec). After successive washes, positively selected CD11b+ microglia and ACSA-2+ astrocytes were eluted.

Lipidomics

Cell isolation and subsequent lipidomic assay was performed on pooled samples obtained from 3 male mice (10-14 weeks old) of the same genotype (APOE3/APOE4) and treatment group (Sham/ISO) at 7 d after cessation. Briefly, total lipids were extracted using the MTBE protocol and analyzed by liquid chromatography coupled with tandem mass spectrometry (LC-MS/MS). The LC-MS/MS analyses were conducted on an Ultimate 3000 Ultra High-Performance Liquid Chromatograph coupled to a Thermo TSQ Altis Triple Quadrupole Mass Spectrometer (Thermo Scientific, San Jose, CA). Chromatographic separation was achieved using an ACQUITY Amide BEH column (1.7 μm, 2.1 × 100 mm; Waters, Milford, MA) maintained at 45 °C. The mobile phases consisted of solvent A (ACN/H_2O, 95:5, v/v) and solvent B (ACN/H_2O, 50:50, v/v), each containing 10 mM ammonium acetate. The gradient program was run at 0.6 mL/min with the following profile: 0.1-20% B over 2 min, 20-80% B over 3 min, 80% to 0.1% B in 0.1 min, followed by equilibration at 0.1% B for 2.9 min. The total run time was 8.0 min, with a 2 μL injection volume. The autosampler was maintained at 7 °C. Electrospray ionization was performed in both positive and negative ion modes, and mass spectrometric detection employed SRM mode. ESI source settings were spraying voltage of +3500 V (positive mode) and −2500 V (negative mode), sheath gas (Arb) = 60, auxiliary gas (Arb) = 15, sweep gas (Arb) = 1, and ion transfer tube temperature of 380 °C. Nitrogen was used as the nebulizer gas and argon as the collision gas (1.5 mTorr). The vaporizer temperature was 350 °C. Collision energies and RF lens voltages were optimized for each lipid class reference standard.

***In vivo* electrophysiological recording and data analysis**

Animals were anesthetized by 2% ISO. To prepare for recordings from the mitral cell layer (MCL) on the medial side of each OB of awake mice, the craniotomy was made on the dorsal skull with the following coordinates: 5.50 mm anterior from Bregma and 0.30 mm lateral from the midline. A metal head-plate with a 4.2-mm round opening (Models 1 and 10; Neurotar, Helsinki, Finland) was attached to the skull with Metabond. One week after surgery, mice were trained for habituation to head fixation in head-fixed treadmill apparatus (Neurotar, Helsinki, Finland) for 15

min/day in 5 consecutive days. On day 12 after headplate implantation, animals received a single 2 h ISO exposure as the experimental treatment. ISO was delivered at 5% for induction and 2% for maintenance with a room air flow rate of 75 ml/min. The depth of anesthesia was confirmed by the loss of hind paw withdrawal reflex tested every 15 minutes. This paradigm isolates the effects of ISO anesthesia following prior cranial surgery required for recording. Groups compared include APOE3/Sham, APOE4/Sham, APOE3/ISO, APOE4/ISO.

To verify the position of the recording sites, probes were coated with two different dyes: Dil-DilC18(3) (red) for the left OB and DiO-DiOC18(3) (green) (Invitrogen) for the right OB. Electrophysiological signals were detected by the neural probe and passed through a digital head stage to a 32-channel amplifier (Plexon DigiAmp, Plexon, TX, USA), where they were sampled at 1 kHz and bandpass filtered at 300-7,500 Hz for spikes and at 0.1-200 Hz for local field potential (LFP) before being digitally sampled at 40 kHz by a Plexon Omniplex recording system (Plexon, Dallas, TX).

Patch clamp recording in brain slices and data analysis

For patch-clamp experiments, mice were assigned to Sham/OP or ISO/OP cohorts. Both cohorts underwent laparotomy under ISO delivered in room air. Sham/OP mice were allowed to recover shortly after completion of the laparotomy, whereas ISO/OP mice were maintained under 2% isoflurane to complete a total exposure time of 2 h. On day 7 after treatment, animals were deeply anesthetized with ISO using an open-drop method prior to decapitation. Horizontal OB and coronal brain sections were cut using a VT1200S vibratome (Leica Microsystems, Germany) in ice-cold, oxygenated (95% O_2/5% CO_2) N-Methyl-D-glucamine (NMDG)-based artificial cerebrospinal fluid (aCSF) containing (in mM): 90 NMDG, 2.5 KCl, 1.2 NaH2PO4, 30 NaHCO3, 25 glucose, 20 NaHEPES, 5 Sodium ascorbate, 2 Thiourea, 3 Sodium pyruvate, 10 MgSO4, 0.5 CaCl2 (pH 7.40, 300 mOsm). After 30 min of incubation in NMDG aCSF at 30°C, slices were then transferred to HEPES-based aCSF at RT (room temperature) until they were used for recordings. HEPES-based aCSF was continuously bubbled with 95% O2-5% CO2 and had the composition (in mM): 92 NaCl, 2.5 KCl, 1.2 NaH2PO4, 30 NaHCO3, 25 glucose, 20 NaHEPES, 2 Sodium ascorbate, 2 Thiourea, 3 Sodium pyruvate, 2 MgSO4, 2 CaCl2 (pH 7.40, 300 mOsm). During the experiments, slices were perfused at 3 ml/min with recording aCSF, which was equilibrated with 95% O2/5% CO2 and warmed to 30°C and had the following composition (in mM): 125 NaCl, 2.5 KCl, 1.25 NaH2PO4, 25 NaHCO3, 10 glucose, 2 MgSO4, 2 CaCl2 (pH 7.40, 300 mOsm).

Whole-cell patch-clamp recordings were performed from neurons in OB or HI slices visualized with an Eclipse FN1 fixed-stage upright microscope (Nikon, Japan) equipped with near-

infrared differential interference contrast optics. External tufted cells in the OB slices or pyramidal neurons in the CA1 region of HI slices were preselected for recording based on their soma location, sizes and shapes as well as the projection patterns of their primary dendrites. Cell identities were further verified by electrophysiological signatures and post hoc morphological reconstruction with histocytochemistry. Biocytin (0.2%) was included in the patch pipette internal solution to fill the recorded neurons thus enable post hoc visualization of their somatodendritic architectures. Electrophysiological signals were acquired using the MultiClamp 700B amplifier (Molecular Devices) in current- or voltage-clamp and pClamp Data Acquisition Software (Clampex v11). Data were sampled 2 kHz or 10 kHz and filtered at 5 kHz or 50 kHz in voltage or current clamp, respectively.

Histochemistry for neural probe position verification and neuronal reconstruction

For histological verification of the 32-channel neural probe positioning in the OB, the mouse brain was dissected immediately after *in vivo* recording and fixed in 4% paraformaldehyde overnight at 4°C. The OB tissue was cut into 150 μm thick coronal sections using a Compresstome® Vibrating Microtomes (VF-510-0Z, Precisionary Instruments, Ashland, MA). Then brain sections were washed in 0.1 M phosphate buffer (PB) for 3 times (5 minutes/each) at room temperature before being incubated in 0.25% Triton X-100 in 0.1 M PB for 1 hour at room temperature for permeabilization. After 3 times (5 minutes/each) wash with 0.1 M PB, brain sections were stained with 4,6-diamidino-2-phenylindole (DAPI) (1:200 dilution) for 10 minutes to label cell nuclei at room temperature and protected from light. DAPI binding was terminated by another 3 times (5 minutes/each) wash with 0.1 M PB before brain sections were mounted onto microscope glass slides using anti-fade mounting medium.

For morphological reconstruction of the patch clamp recorded neurons in the OB or HI, brain slices with biocytin (0.2%, w/v)-filled cells were immediately kept in 4% paraformaldehyde at 4°C overnight. After three (5 min each) washes with 0.05 m phosphate-buffered saline (PBS), slices were incubated in a blocker solution on a shaker for 1 h. Blocker solution was made by 0.05 m PBS with the addition of bovine serum albumin and Triton X-100 with final concentrations of 1% (w/v) and 0.5% (v/v), respectively. The slices were then transferred to and kept in this blocker solution containing streptavidin-CY3 (1 μg/mL) covered with aluminum foil to prevent light exposure at room temperature on a shaker for 7 h. Following three (5 min each) rinses with 0.05 m PBS to terminate streptavidin-CY3 staining, slices were treated with 0.05 m PBS containing DAPI (5 μg/mL) at room temperature in the dark for 10 min. DAPI staining was terminated by three (5 min each) washes with PBS before slices was wet mounted and cover-slipped with

fluorescence mounting media. Confocal images were acquired using a Zeiss LSM 900 microscope equipped with a 2.5× or 40× oil-immersion objective. Z-stacks were collected with a step size of 1 μm and an XY resolution of 0.21 μm/pixel.

Neurological assessment

Odor memory (OM) test: To evaluate olfactory learning and memory, mice were singly housed overnight and subjected to a previously described OM protocol [1, 2]. A sterile, 6-inch cotton-tipped wooden applicator was introduced through a cage-lid port, allowing the animal 30 minutes of familiarization. Each mouse was provided with a new applicator. For odor presentation, cinnamon powder (McCormick; 100 ng/ml in water) was freshly prepared and stored in airtight vials. During the first trial (T1), the applicator tip was briefly immersed in the odor solution (2 s) and inserted ~2.5 cm into the cage. Sniffing behavior, defined as nose orientation toward the applicator within 2 cm, was recorded over 5 minutes. Following a 60-minute interval, a second trial (T2) was conducted identically. Memory retention was quantified as the [T2/T1] × 100 sniffing ratio, with reduced exploration in T2 reflecting recognition of the familiar odor.

Buried food (BF) test: To assess olfactory function, BF was performed to measure the animal's ability to detect and retrieve a familiar food reward hidden under bedding as described in previous papers [1, 3]. Mice were individually housed with free access to water but food-deprived for 24 h to increase motivation. The evening prior to testing, each subject received a mini cookie in its home cage for odor familiarization, and consumption was confirmed the next morning. On the test day, mice were acclimated for 10 min in a clean cage (46 × 23.5 × 20 cm) containing 3 cm of fresh bedding, then returned to their home cage. A cookie (~1 g) was buried 2-3 cm beneath the bedding in a randomly chosen corner, and the mouse was reintroduced. The latency to locate and begin eating the buried cookie was recorded, with trials ending after 15 min if unsuccessful.

Nest building (NB) test: The NB is a widely used non-invasive behavioral assay, was employed to assess rodent well-being, motivation, and cognitive function [4]. Mice were singly housed in standard cages containing wood chip bedding. Each cage was supplied with a pre-weighed pressed cotton square (nestlet) as the sole nesting material. Animals were left undisturbed overnight (~12 h), after which nest quality was scored the next morning on a standardized 5-point scale, ranging from 0 (nestlet untouched) to 5 (well-formed dome). Remaining nesting material was weighed to quantify usage, and nests were photographed for documentation. Poor performance in this task is indicative of deficits in executive function, motor planning, or motivation.

Grip strength (GS) test: This test in mice is designed to measure neuromuscular function, particularly forelimb and/or hindlimb strength using a digital grip strength meter (Ugo Basile) based on our established protocols[3]. Briefly, the forelimb GS of both paws was assessed by placing the mouse on a mesh wire grid connected to the device's force transducer. Once the mouse secured a firm grip, it was gently held by the tail and slowly pulled away from the grid. The maximum force exerted on the mesh wire grid was recorded, with each mouse undergoing an average of 10 daily trials. Final GS values were normalized to body weight for comparison between groups.

Rotarod (RT) test: Locomotor performance was assessed using the Rotarod (Harvard Apparatus) following established protocols[5]. The device was programmed to accelerate from 4 to 40 rpm over 90 s, with each trial capped at 300 s. Mice were positioned on the rotating rod prior to start, and the latency to fall was recorded. For each animal, the mean latency across five trials was calculated and used for group comparisons.

Y maze (YM): The YM test was used to assess hippocampus-dependent spatial working memory in mice, following established methods [3]. The apparatus (Stoelting Co.) consisted of three identical arms (A, B, C). Each trial began with the mouse placed at a randomly chosen start arm, after which it was allowed to explore freely for 5 min. Arm entries were recorded, and a spontaneous alternation was defined as consecutive entries into three different arms. Alternation percentage was calculated as: (total alternations ÷ [total arm entries – 2]) × 100. Mice achieving scores above 50%, the chance level, were considered to have intact spatial working memory.

Open field (OF) test: Spontaneous locomotor activity was assessed in the OF test under dim red illumination [3, 6]. Mice were placed in a corner of the 22.5 × 22.5 cm chamber, facing the wall, and allowed to explore for 5 min. Movements were tracked using the Any-maze software, which quantified parameters such as total distance traveled, mean velocity, immobility duration, and percentage of time spent in the central zone.

Novel object recognition (NOR) test: Non-spatial recognition memory was assessed using the NOR test, which exploits the rodent's innate preference for novelty [3]. On day 1, mice were habituated to the open-field arena for 5 min. On day 2, they were exposed to two identical objects, and exploration time for each was recorded with ANY-maze software (Stoelting) until a total of 30 s was reached (maximum session time: 40 min). On day 3, one familiar object was replaced with a novel one, and testing proceeded under the same conditions. Recognition memory was considered intact when mice spent >15 s exploring the novel object.

RNAseq analysis

We used STAR aligner version 2.7.5 to map RNAseq reads to the mouse reference genome (GRCm39) with GENCODE gene annotation (version M33) [7]. We quantified gene and isoform expression at the transcripts per million (TPM) level using RSEM 1.3.3 [8]. We used DESeq2 version 1.42.1 for the differential expression analysis, with false discovery rates (FDR) derived from the Benjamini-Hochberg method [9]. Genes with an FDR of less than 0.05 were considered to be differentially expressed genes (DEGs) and used for downstream pathway enrichment analysis. Gene ontology (GO) analysis was performed with cluster Profiler 4.10.1 using DESeq2 output [10].

Additional statistical and experimental design details

Distinct animal cohorts were used for different experimental modalities unless otherwise specified. Because tissue collection required euthanasia, data obtained from separate tissue, cellular, electrophysiological, and molecular experiments represent independent biological samples. RNA sequencing was performed using tissue collected from mice after completion of behavioral testing, whereas electrophysiological recordings and most other assays were conducted in independent cohorts.

Sample sizes were based on prior studies and pilot experiments using comparable experimental designs and endpoints. Group sizes were selected to detect biologically relevant differences and were generally sufficient to detect medium-to-large effects at $\alpha = 0.05$ according to Cohen’s effect-size conventions. No formal prospective power calculation was performed because the study incorporated multiple exploratory and mechanistically distinct experimental modalities.

Animals were included according to the prespecified genotype, age, sex, treatment, and experimental-completion criteria described in the relevant Methods subsections. All animals that completed the experimental protocol were included in the analysis. No animals or data points were excluded based on the observed outcome. Humane endpoints were established a priori in accordance with institutional IACUC guidelines; no animals met these criteria during the study. Outcome measures included performance across multiple behavioral assessments, electrophysiological properties such as membrane resistance, resting membrane potential, synaptic transmission, and current injection-evoked firing, and molecular, transcriptomic, and lipidomic profiles of brain tissues and isolated cell populations. Individual outcome measures are described in the corresponding Methods subsections and summarized in the experimental workflow schematic (Supplementary Fig. 1). Given the exploratory, multimodal design of the study, no single primary outcome measure was predefined.

For each experiment, the specific statistical model, factors, post hoc comparisons, multiple-testing corrections, and sample sizes are reported in the corresponding figure legends or assay-specific Methods subsections. Statistical significance was defined as $p < 0.05$ unless otherwise specified. For high-dimensional analyses, adjusted p values or false-discovery-rate thresholds were applied as described for each dataset.

References

1. Liu X, Lei Z, Gilhooly D, He J, Li Y, Ritzel RM, Li H, Wu LJ, Liu S, Wu J: **Traumatic brain injury-induced inflammatory changes in the olfactory bulb disrupt neuronal networks leading to olfactory dysfunction.** *Brain Behav Immun* 2023, **114:**22–45.
2. Tillerson JL, Caudle WM, Parent JM, Gong C, Schallert T, Miller GW: **Olfactory discrimination deficits in mice lacking the dopamine transporter or the D2 dopamine receptor.** *Behav Brain Res* 2006, **172:**97–105.
3. Li Y, Uzun C, Islam ST, Krishnamachary B, Lee H, Wang Z, Li H, Liu S, Wu J: **Age-dependent Transcriptional and Circuit Alterations in the brain Underlie Post-Anesthesia Neurobehavioral Dysfunction.** *Aging Dis* 2025.
4. Deacon R: **Assessing burrowing, nest construction, and hoarding in mice.** *J Vis Exp* 2012**:**e2607.
5. Ritzel RM, Li Y, Jiao Y, Doran SJ, Khan N, Henry RJ, Brunner K, Loane DJ, Faden AI, Szeto GL, Wu J: **Bi-directional neuro-immune dysfunction after chronic experimental brain injury.** *J Neuroinflammation* 2024, **21:**83.
6. Li Y, Ritzel RM, Lei Z, Cao T, He J, Faden AI, Wu J: **Sexual dimorphism in neurological function after SCI is associated with disrupted neuroinflammation in both injured spinal cord and brain.** *Brain Behav Immun* 2022, **101:**1–22.
7. Dobin A, Davis CA, Schlesinger F, Drenkow J, Zaleski C, Jha S, Batut P, Chaisson M, Gingeras TR: **STAR: ultrafast universal RNA-seq aligner.** *Bioinformatics* 2013, **29:**15–21.
8. Li B, Dewey CN: **RSEM: accurate transcript quantification from RNA-Seq data with or without a reference genome.** *BMC Bioinformatics* 2011, **12:**323.
9. Love MI, Huber W, Anders S: **Moderated estimation of fold change and dispersion for RNA-seq data with DESeq2.** *Genome Biol* 2014, **15:**550.
10. Wu T, Hu E, Xu S, Chen M, Guo P, Dai Z, Feng T, Zhou L, Tang W, Zhan L, et al: **clusterProfiler 4.0: A universal enrichment tool for interpreting omics data.** *Innovation (Camb)* 2021, **2:**100141.
