# Supplementary material for: Isoflurane and Surgery Aggravate APOE4-Dependent Lipid Dysregulation, Neural Dysfunction, and Neurological Impairment in Male Mice
Source: Cells. 2026 Nov 9;15(21):2038. doi: 10.3390/cells15212038 (PMC13762270; doi:10.3390/cells15212038)
Supplement: Supplementary file 1 [file cells-15-02038-s001.zip › Supple Materials 2_Additional Figures 11-2-2026 yl.pdf]

Isoflurane and surgery aggravate APOE4-dependent lipid dysregulation, neural dysfunction, and neurological impairment in male mice

Yun Li[1a], Yuanyuan Ji[2a], Cosar Uzun[3], Syed Taufiqul Islam[3], Meigeng Hu[3], Dan Zhao[3], Yaping Li,[3] Hangnoh Lee[4], Zihui Wang[1], Hui Li[1], Jace W. Jones[2], Shaolin Liu[3]*, Junfang Wu[1,5]*

[1]Department of Anesthesiology and Center for Shock, Trauma and Anesthesiology Research (STAR), University of Maryland School of Medicine, Baltimore, MD 21201, USA

[2]Department of Pharmaceutical Sciences, University of Maryland School of Pharmacy, Baltimore, MD 21201, USA

[3]Center for Neurological Disease Research, Department of Physiology and Pharmacology, Department of Biomedical Sciences, University of Georgia College of Veterinary Medicine, Athens, GA 30602, USA

[4]Department of Medicine, University of Maryland School of Medicine, Baltimore, MD 21201, USA

[5]University of Maryland—Medicine Institute for Neuroscience Discovery (UM-MIND), University of Maryland School of Medicine, Baltimore, MD 21201, USA

[a] Contributed equally to the manuscript.

*Correspondence: Junfang Wu, Email: junfang.wu@som.umaryland.edu or Shaolin Liu, Email: shaolin.liu@uga.edu

Supplemental Materials

Supplemental Figures (17) and Tables (4)

Supplemental Figure Legends

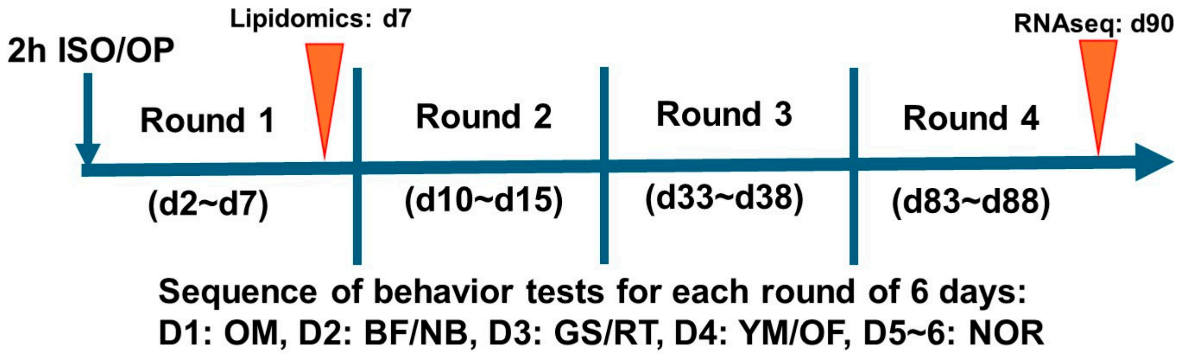

Figure S1 Schematic illustration of the experimental workflow for a battery of neurological behavioral tests and timing of tissue collection. In addition to the cohort of mice used for evaluation of neurological functions, a separate age-matched cohort underwent the same treatment and was used for lipidomics. At 90d after cessation, the hippocampus and olfactory bulb regions were extracted from the behavioral study cohort and used for RNA sequencing (RNAseq). OM: odor memory, BF: buried food, NB: nest building, GS: grip strength, RT: rotarod, YM: Y maze, OF: open field, NOR: novel object recognition.

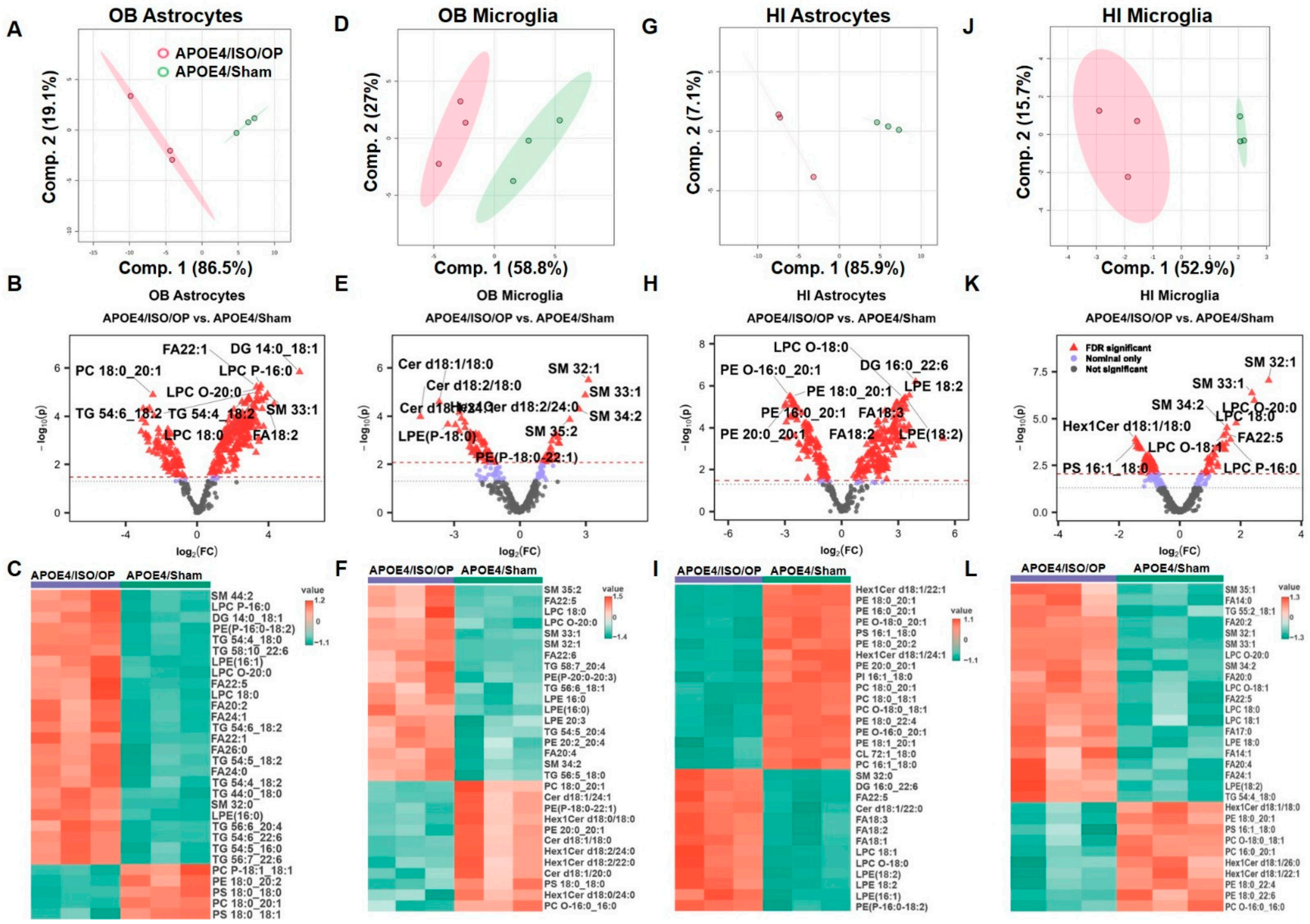

Figure S2 Isoflurane (ISO) exposure induces lipid dysregulation in the olfactory bulb (OB) and hippocampus (HI) astrocytes and microglia of APOE4 mice. **A-C** OB astrocytes, **D-F** OB microglia, **G-I** hippocampal astrocytes, and **J-L** hippocampal microglia from APOE4/ISO/OP vs. APOE4/Sham mice at 7 days post-exposure. In each set, Partial least squares discriminant analysis (PLS-DA) illustrates sample clustering. Volcano plots display $\log_2$ fold changes and nominal p-values, with red triangles indicating lipids meeting the Benjamini-Hochberg false discovery rate (FDR)-adjusted significance threshold (adjusted $p \leq 0.05$), purple points indicating nominal significance only ($p < 0.05$), and grey points indicating non-significant lipids. The red dotted horizontal line represents the nominal p-value cutoff corresponding to the FDR-adjusted significance threshold for each comparison. Heatmaps show the top 30 lipids selected by nominal Student's t-test p-values for exploratory visualization. n = 3 pooled biological samples/group, each comprising three male mice (9 mice/group).

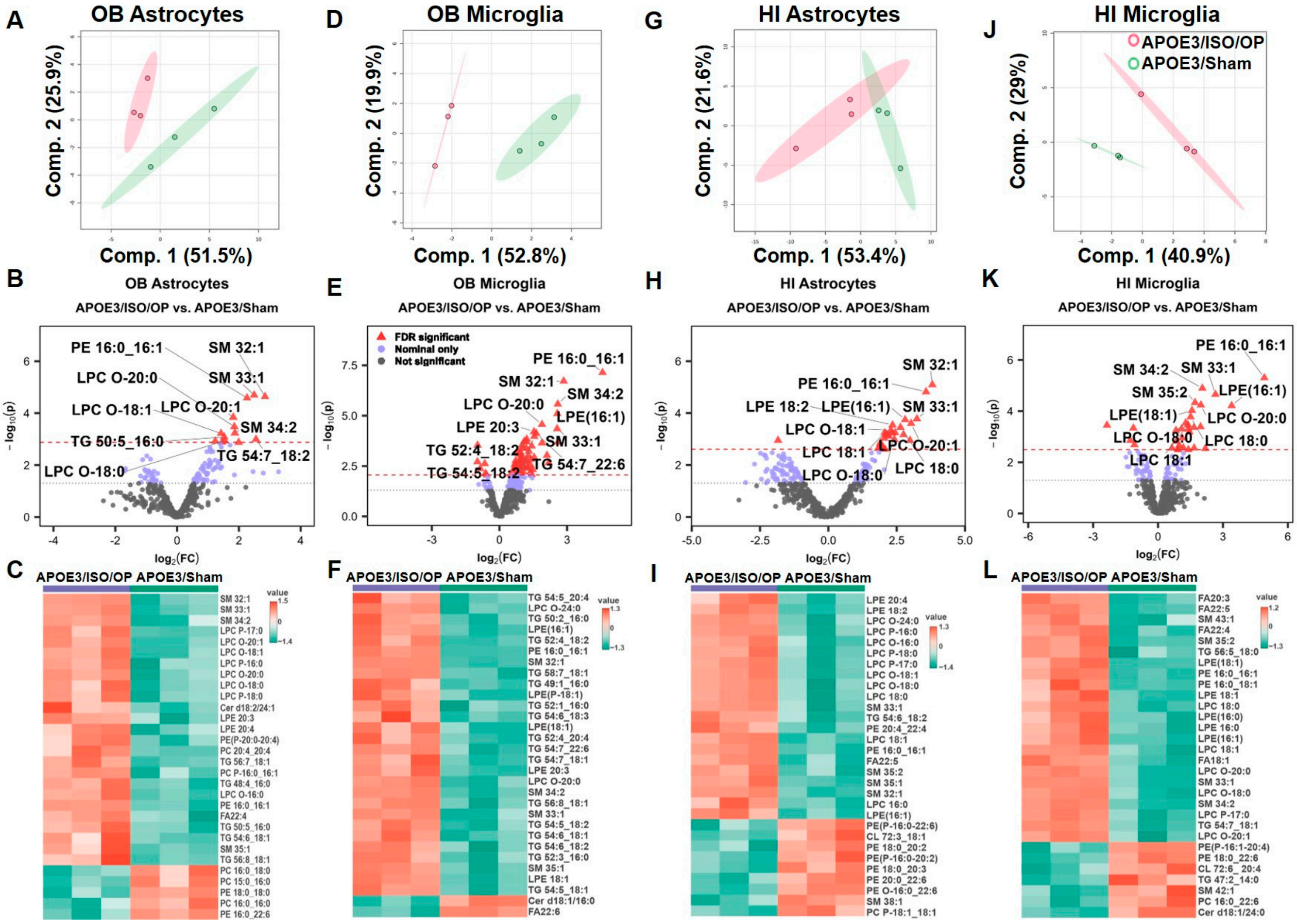

Figure S3 Isoflurane (ISO) exposure induces olfactory bulb (OB) and hippocampus (HI) glial lipid dysregulation, even in APOE3 mice. **A-C** OB astrocytes, **D-F** OB microglia, **G-I** hippocampal astrocytes, and **J-L** hippocampal microglia from APOE3/ISO/OP vs. APOE3/Sham mice at 7 days post-exposure. In each set, Partial least squares discriminant analysis (PLS-DA) illustrates sample clustering. Volcano plots display $\log_2$ fold changes and nominal p-values, with red triangles indicating lipids meeting the Benjamini-Hochberg false discovery rate (FDR)-adjusted significance threshold (adjusted $p \leq 0.05$), purple points indicating nominal significance only ($p < 0.05$), and grey points indicating non-significant lipids. The red dotted horizontal line represents the nominal p-value cutoff corresponding to the FDR-adjusted significance threshold for each comparison. Heatmaps show the top 30 lipids selected by nominal Student's t-test p-values for exploratory visualization. n = 3 pooled biological samples/group, each comprising three male mice (9 mice/group).

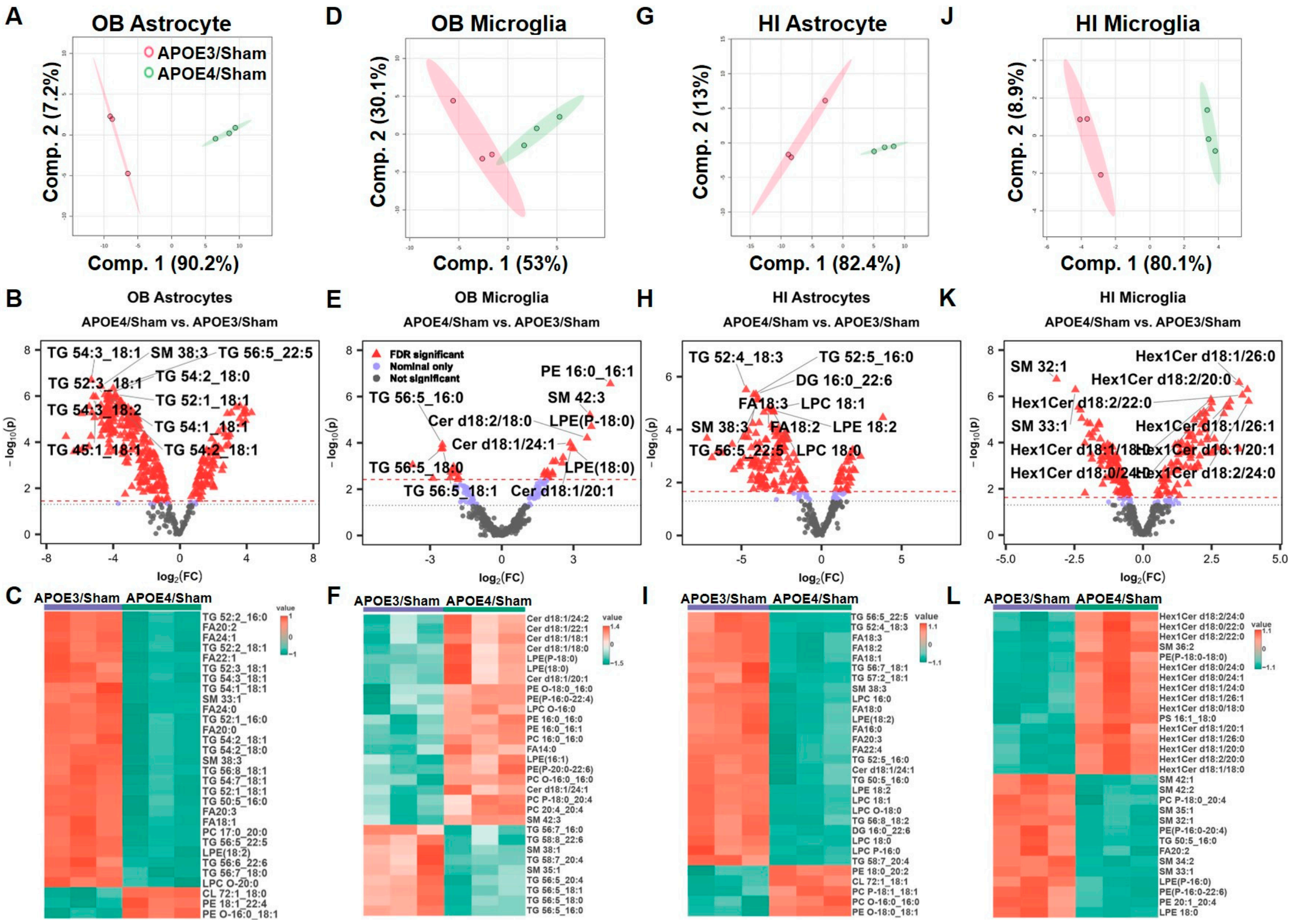

Figure S4 Humanized APOE4 mice display distinct baseline glial lipid profiles. A-C OB astrocytes, **D-F** OB microglia, **G-I** hippocampal astrocytes, and **J-L** hippocampal microglia from APOE4/Sham vs. APOE3/Sham mice. In each set, Partial least squares discriminant analysis (PLS-DA) illustrates sample clustering. Volcano plots display $\log_2$ fold changes and nominal p-values, with red triangles indicating lipids meeting the Benjamini-Hochberg false discovery rate (FDR)-adjusted significance threshold (adjusted $p \leq 0.05$), purple points indicating nominal significance only ($p < 0.05$), and grey points indicating non-significant lipids. The red dotted horizontal line represents the nominal p-value cutoff corresponding to the FDR-adjusted significance threshold for each comparison. Heatmaps show the top 30 lipids selected by nominal Student's t-test p-values for exploratory visualization. n = 3 pooled biological samples/group, each comprising three male mice (9 mice/group).

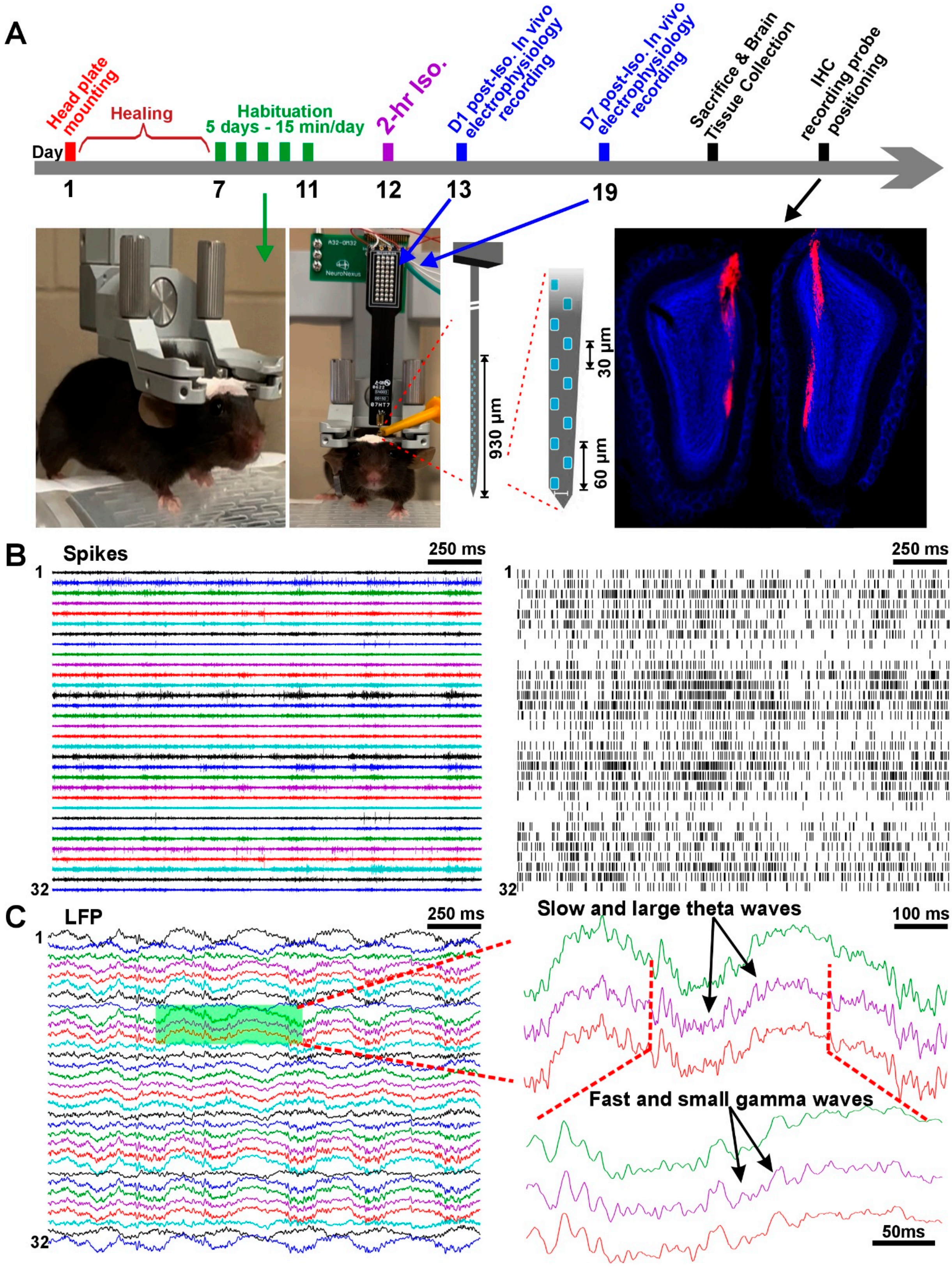
A
Head plate mounting
Healing
Habituation
5 days - 15 min/day
2-hr Iso.
D1 post-Iso. In vivo electrophysiology recording
D7 post-Iso. In vivo electrophysiology recording
Sacrifice & Brain Tissue Collection
IHC recording probe positioning
Day
1
7
11
12
13
19
30 µm
930 µm
60 µm
B
Spikes
250 ms
1
32
C
LFP
250 ms
Slow and large theta waves
100 ms
Fast and small gamma waves
50ms

Figure S5 *In vivo* electrophysiological recordings in awake and head-fixed mice. **A** schematic illustration of the experimental design. Photos showing recording from an awake and head fixed mouse using 32-channel probe, confocal image verifying the probe penetration path in the OB mitral cell layer. **B** typical traces of spike activities (Left) and raster graph showing detected spikes from the left trace (Right). **C** typical local field potential (LFP) traces showing the neural oscillations (Left) and blown-up traces showing the detection of slow and large theta (top) or fast and small gamma oscillatory waves (bottom). IHC: Immunohistochemistry, D1 Post-Iso.: Day 1 post-isoflurane, D7 Post-Iso.: Day 7 post-isoflurane.

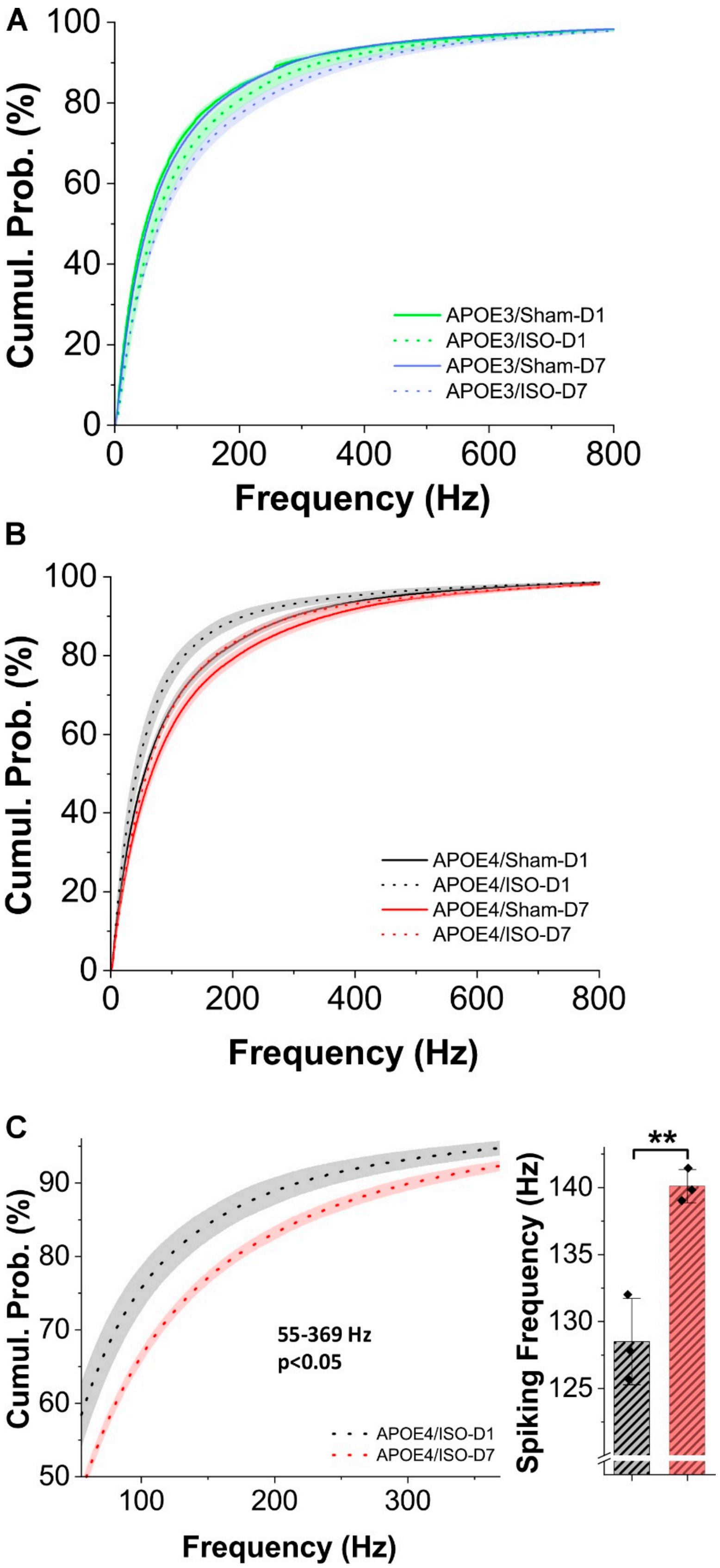
A
Cumul. Prob. (%)
Frequency (Hz)
APOE3/Sham-D1
APOE3/ISO-D1
APOE3/Sham-D7
APOE3/ISO-D7
B
Cumul. Prob. (%)
Frequency (Hz)
APOE4/Sham-D1
APOE4/ISO-D1
APOE4/Sham-D7
APOE4/ISO-D7
C
Cumul. Prob. (%)
55-369 Hz
p<0.05
APOE4/ISO-D1
APOE4/ISO-D7
Frequency (Hz)
Spiking Frequency (Hz)
**

Figure S6 Comparison of isoflurane (ISO) effects on neuronal excitability between post-ISO day 1 (D1) and day 7 (D7). A &B, Comparison of the cumulative probability of spiking frequency in APOE3 (A) and APOE4 mice (B) from the sham (solid green vs blue curve in APOE3 and solid black vs red curve in APOE4) or ISO-exposed (dashed green vs blue curve in APOE3 and dashed black vs red curve in APOE4) group between D1 and D7 using the number of animals as the sample size, with the false discovery rate (FDR) correction applied. **C**, An enlarged view of B within the frequency range 55-369 Hz with statistically significant differences for the comparisons between APOE4/ISO-D7 and APOE4/ISO-D1 groups and a bar graph representing the animal level comparison of the averaged spiking frequency within the frequency range shown in the left using a two-sample t-test. **$p<0.01$.

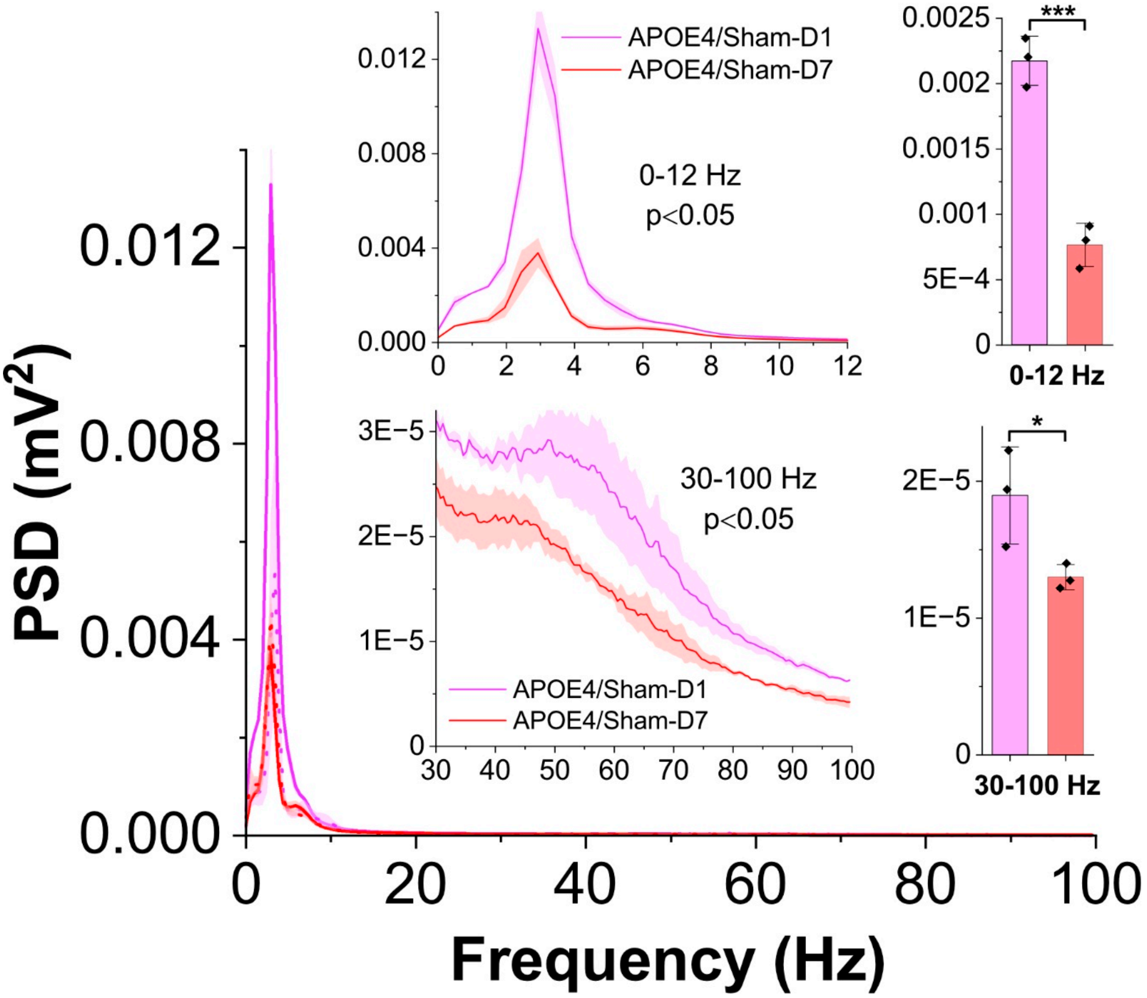

Figure S7 Comparison of isoflurane (ISO) effects on local field potential (LFP) oscillations between post-treatment day 1 (D1) and day 7 (D7) in APOE4 mice. The primary line graph compares the power spectral density (PSD) of LFP oscillatory activities at the animal level between the post-treatment day 7 and day 1 groups of APOE4 sham mice. The line graph insets are enlarged views of the primary line graph highlighting the frequency ranges (0-12 Hz and 30-100 Hz) with statistical significance. Each curve (solid or dashed) with shaded area presents mean ± SE. Bar graphs represent the averaged PSD within the corresponding frequency ranges from the left line graph insets. The line graphs were compared using the number of animals as the sample size, with the false discovery rate (FDR) correction applied. The bar graphs were analyzed using a two-sample t-test. *$p<0.05$, ***$p<0.001$.

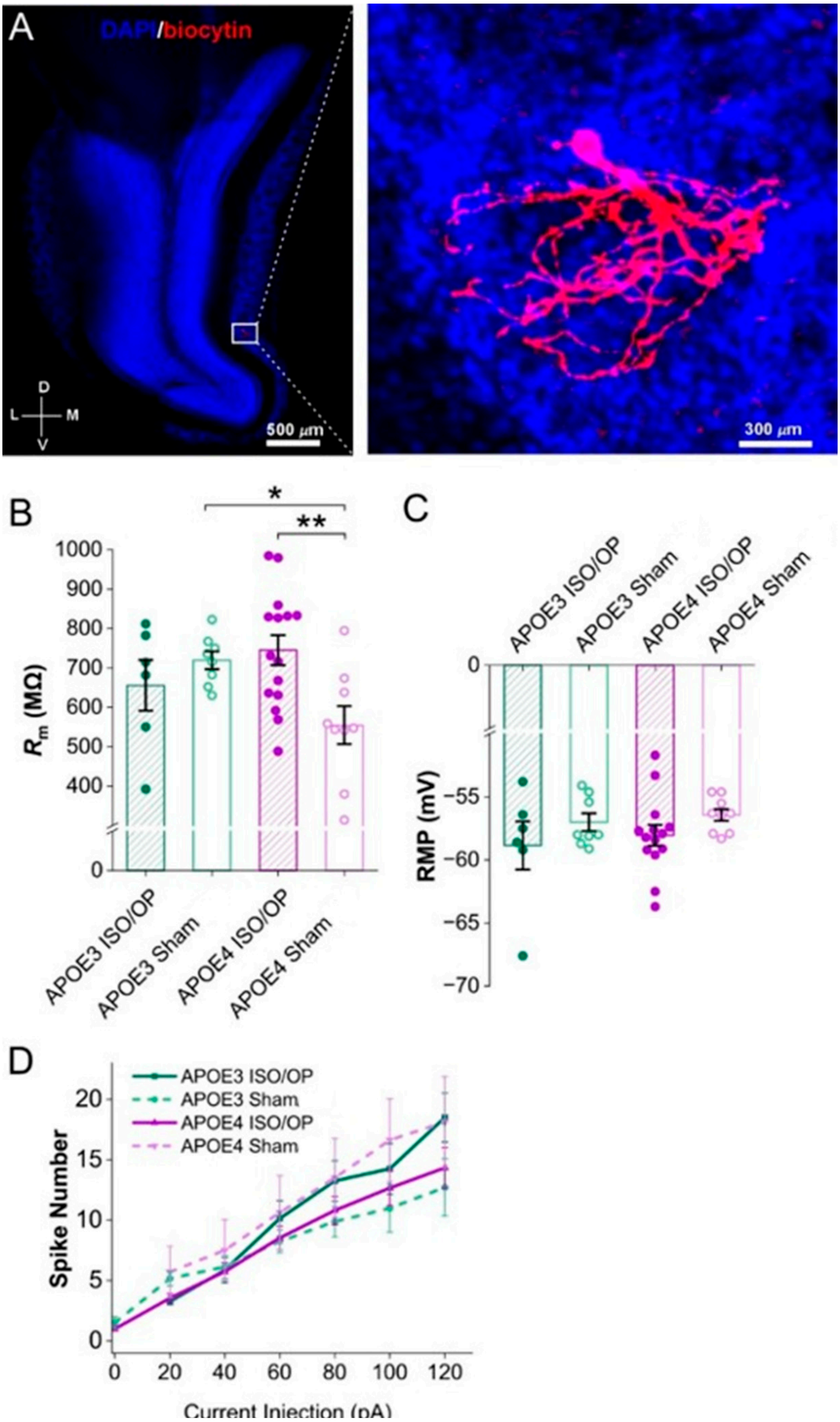

A
DAPI/biocytin
D
L
M
V
500 μm
300 μm
B
*
**
1000
900
800
700
600
500
400
0
R_m (MΩ)
APOE3 ISO/OP
APOE3 Sham
APOE4 ISO/OP
APOE4 Sham
C
APOE3 ISO/OP
APOE3 Sham
APOE4 ISO/OP
APOE4 Sham
0
-55
-60
-65
-70
RMP (mV)
D
APOE3 ISO/OP
APOE3 Sham
APOE4 ISO/OP
APOE4 Sham
Spike Number
0
5
10
15
20
0
20
40
60
80
100
120
Current Injection (pA)

Figure S8 Effects on membrane properties of external tufted cells in the olfactory bulb (OB). **A** Confocal image of a DAPI-counterstained (blue) coronal OB slice with a representative external tufted cell (ETC) filled by biocytin (red) through a patch clamp recording electrode. **B-D** comparisons of membrane resistance (Rm, B), resting membrane potential (RMP, C) and number of spikes evoked by injections of stepped depolarizing currents (200 ms, 20 pA/step, D) in ETCs from four groups of mice: APOE3 with laparotomy (APOE3/Sham/OP) and 2-h isoflurane (APOE3/ISO/OP), APOE4 with laparotomy (APOE4/Sham/OP) and 2-h isoflurane (APOE4/ISO/OP). Data was presented as mean ± SE and analyzed with Two-way ANOVA and Bonferroni comparisons test. *$p<0.05$, **$p<0.01$.

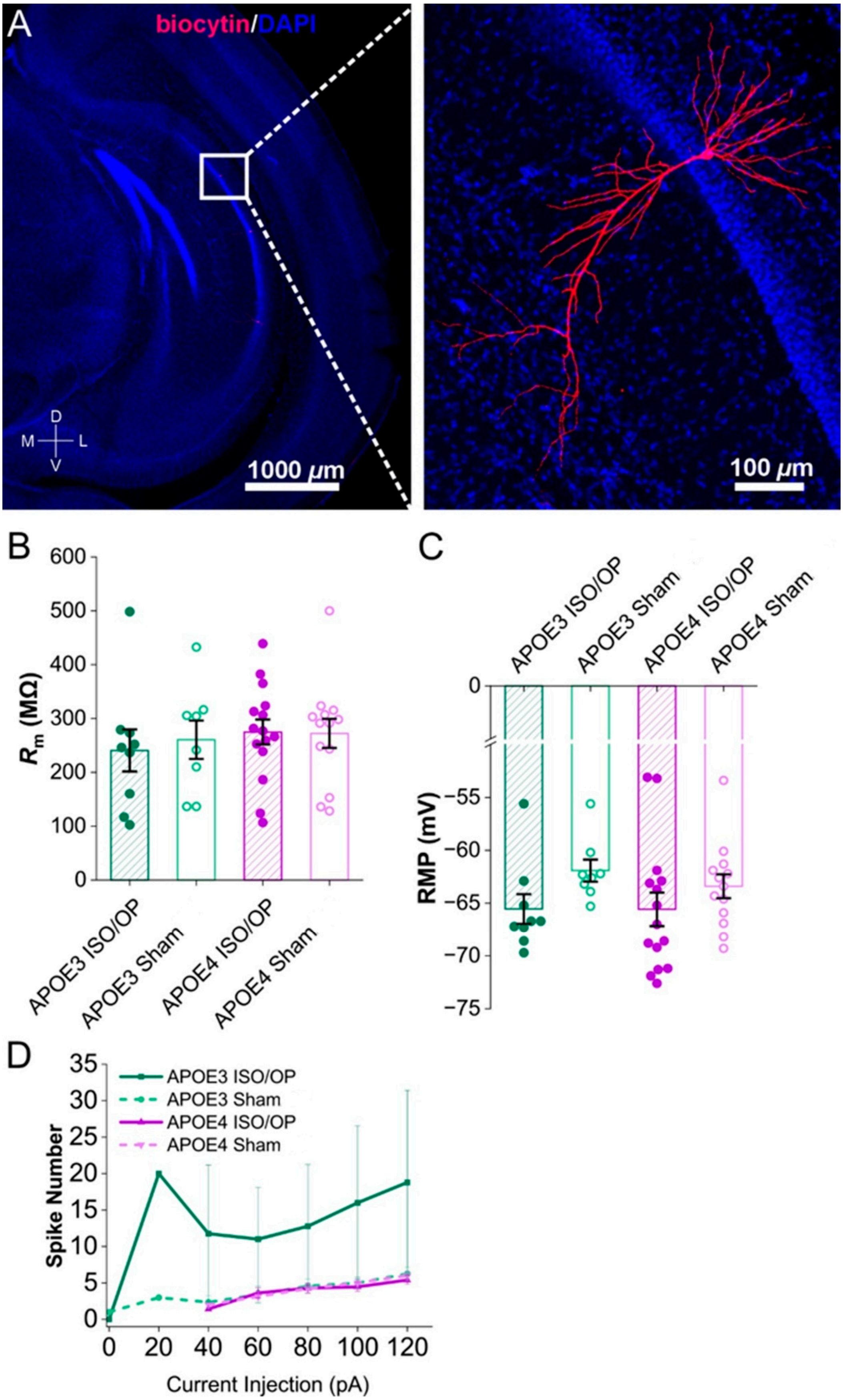

A
biocytin/DAPI
D
M
L
V
1000 μm
100 μm
B
Rm (MΩ)
APOE3 ISO/OP
APOE3 Sham
APOE4 ISO/OP
APOE4 Sham
C
RMP (mV)
D
Spike Number
Current Injection (pA)

Figure S9 Effects on membrane properties of pyramidal neurons in the hippocampus. **A** Confocal image of a DAPI-counterstained (blue) coronal OB slice with a representative pyramidal neuron filled by biocytin (red) through a patch clamp recording electrode. **B-D** Comparisons of membrane resistance (Rm, B), resting membrane potential (RMP, C) and number of spikes evoked by injections of stepped depolarizing currents (200 ms, 20 pA/step, D) in pyramidal neurons from four groups of mice: APOE3 with laparotomy (APOE3/Sham/OP) and 2-h ISO (APOE3/ISO/OP), APOE4 with laparotomy (APOE4/Sham/OP) and 2-h isoflurane (APOE4/ISO/OP). Data was presented as mean ± SE and analyzed with Two-way ANOVA and Bonferroni comparisons test.

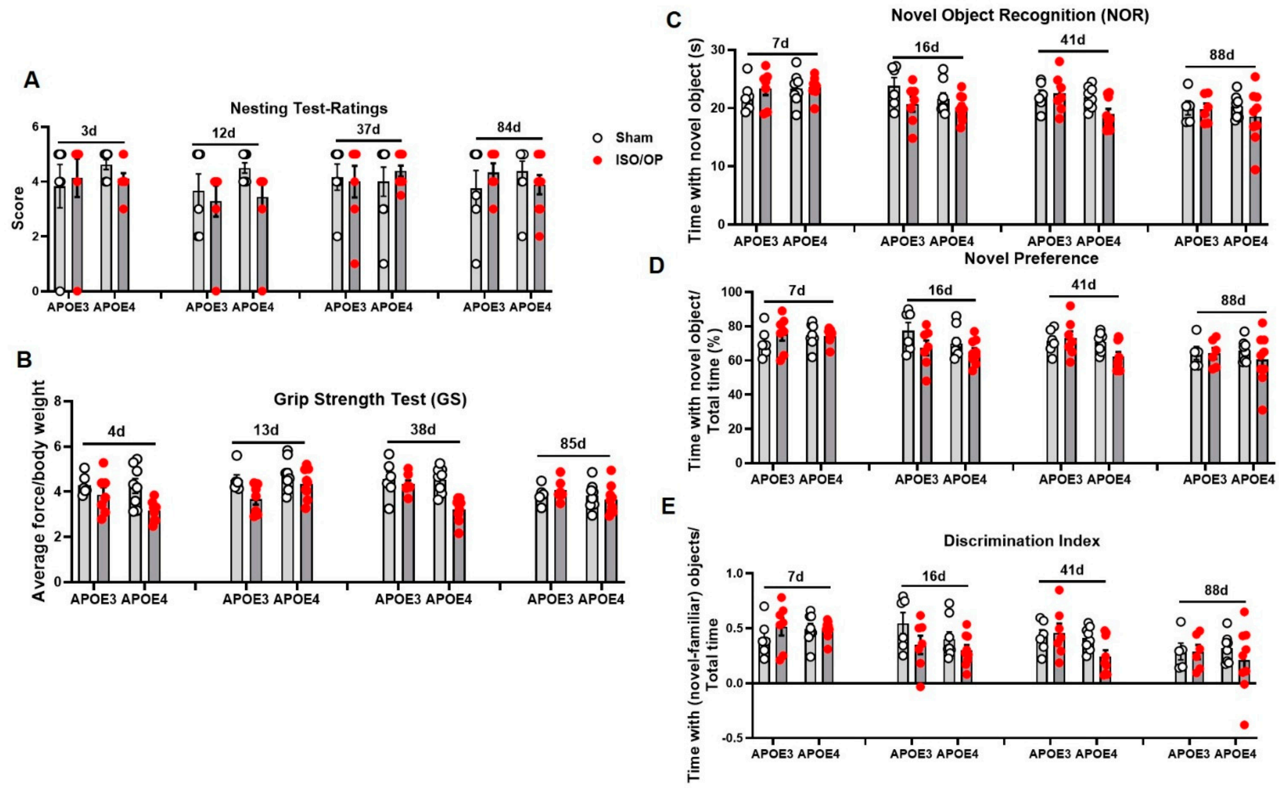

Figure S10 Short term isoflurane (ISO) exposure does not affect the general well-being and neuromuscular functions of APOE4 and APOE3 mice. **A** General-welling was examined by the nest-building (NB) test with quantified nest scores. **B** Neuromuscular function was unaffected by ISO exposure, as evidenced by grip strength (GS) test. **C-E** Non-spatial memory was assessed using the Novel Object Recognition (NOR) test. During the choice phase, the time spent exploring the novel object (C), the animal's preference for the novel object (D), and the discrimination index (E) were recorded. n=6 (APOE3/Sham), 7 (APOE3/ISO/OP), 8 (APOE4/Sham), and 9 (APOE4/ISO/OP). Data was analyzed with Two-way ANOVA and Tukey's multiple comparisons test.

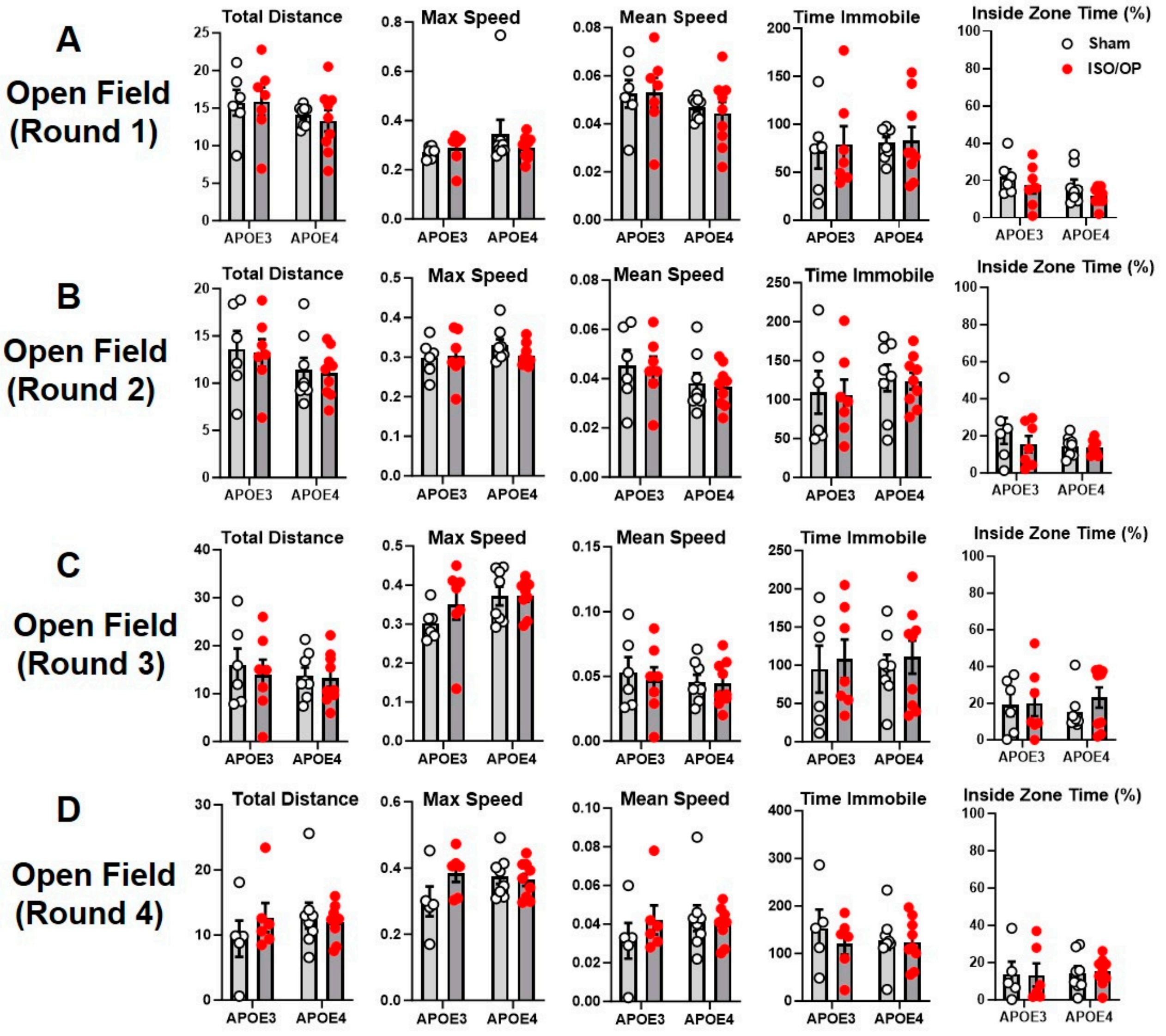

Figure S11 Isoflurane (ISO) does not affect the spontaneous locomotor activity of humanized APOE4 and APOE3 mice. The AnyMaze behavior system was used to record and analyze spontaneous activity in an open field apparatus at round 1 (**A)**, round 2 (**B0**, round 3 (**C)** and round 4 **(D)** after ISO exposure. n=6 (APOE3/Sham), 7 (APOE3/ISO/OP), 8 (APOE4/Sham), and 9 (APOE4/ISO/OP). Data was analyzed with Two-way ANOVA and Tukey’s multiple comparisons test.

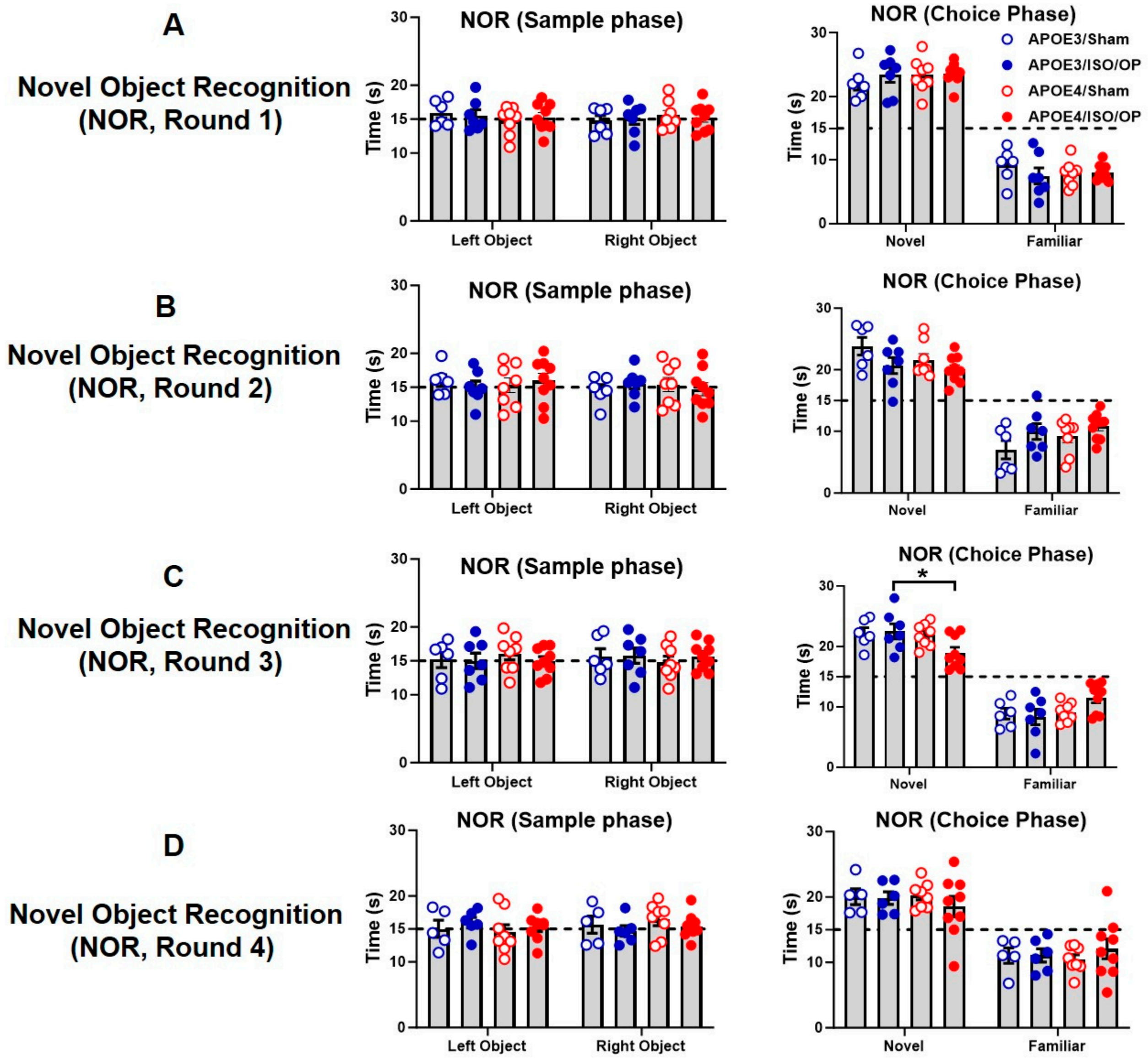

Figure S12 There were no differences between the groups in the sample phase of the novel object recognition (NOR) test. **A-D** A short-term 2h exposure to isoflurane (ISO) with laparotomy did not affect placement preference when exploring the familiar objects but showed a transient impairment on round 3 (C) before recovery on the final round of testing. n=6 (APOE3/Sham), 7 (APOE3/ISO/OP), 8 (APOE4/Sham), and 9 (APOE4/ISO/OP). Data was analyzed with Two-way ANOVA and Tukey's multiple comparisons test.

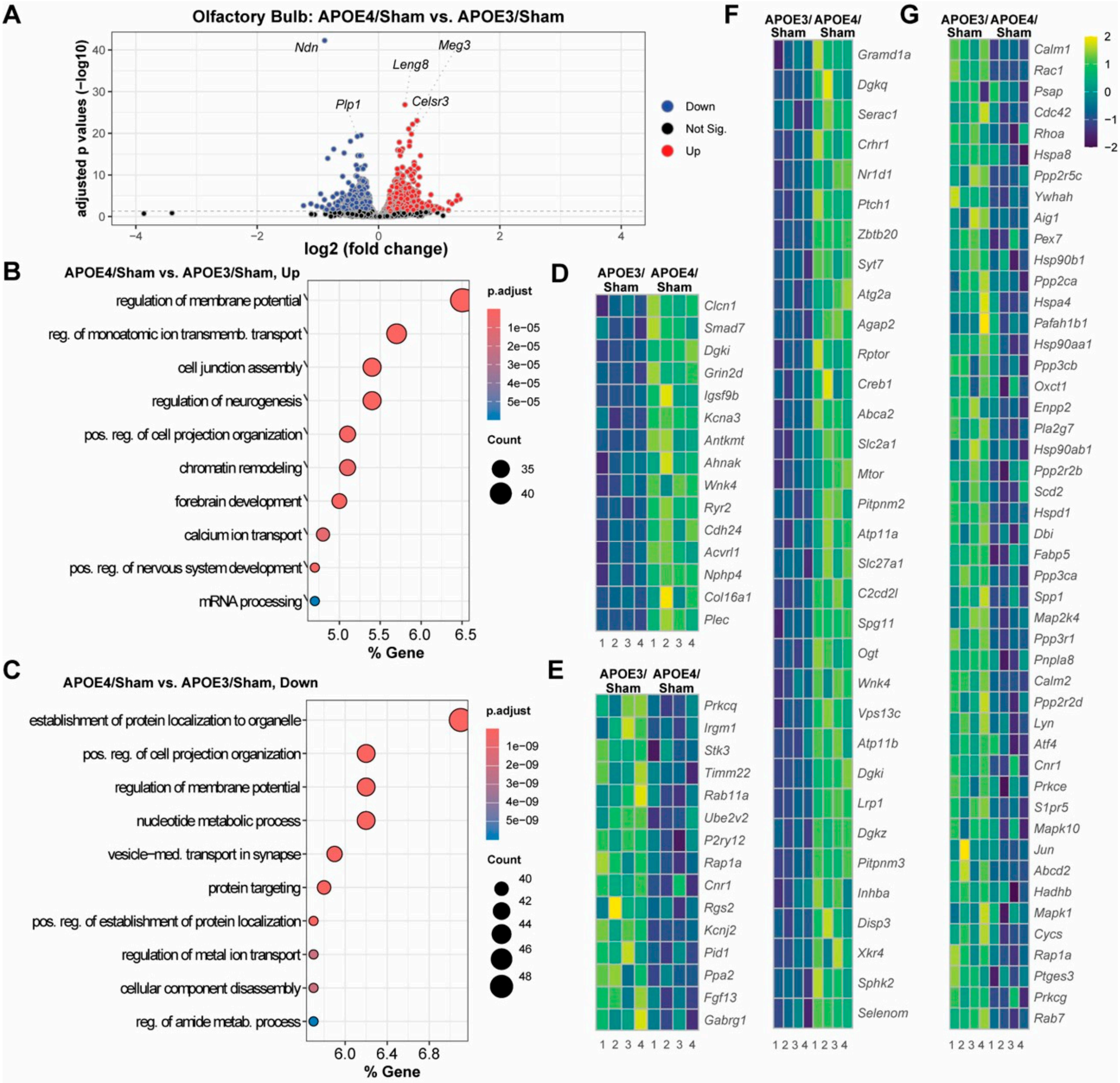

Figure S13 Baseline olfactory bulb transcriptomes differ markedly between APOE4 and APOE3 mice. **A** Volcano plot displaying differentially expressed genes (DEGs) in the APOE4/Sham vs. APOE3/Sham comparison. **B-C** Pathway enrichment analysis of up (B) and downregulated (C) DEGs with Gene Ontology molecular processes. **D-E** Heatmaps displaying DEGs associated with the top three upregulated pathways (D) and top three downregulated pathways (E) identified between APOE4/Sham vs. APOE3/Sham groups. **F** Heatmap of genes involved with lipid metabolism. n=4 mice/group.

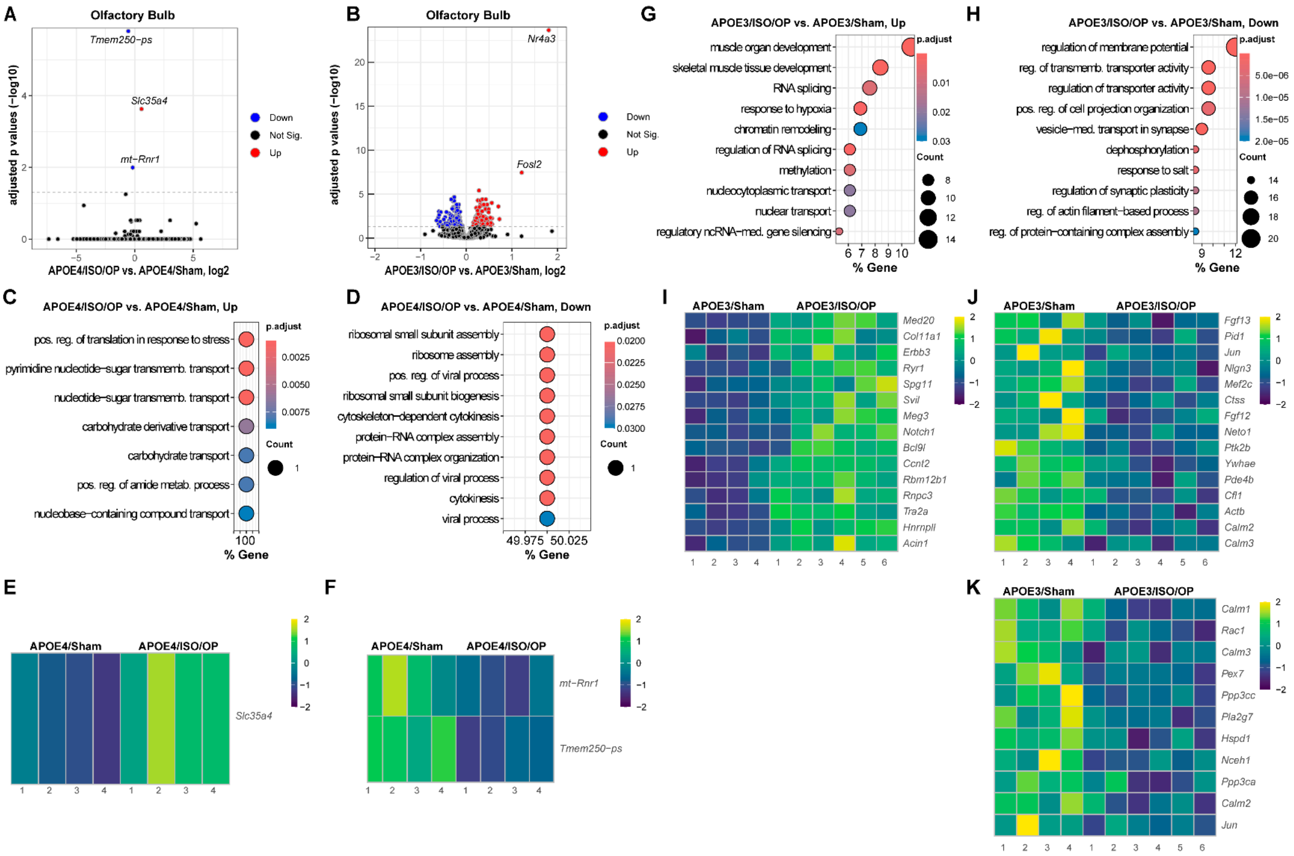

Figure S14 RNAseq reveals molecular alterations in the olfactory bulb (OB) 90d after isoflurane (ISO) exposure. **A-B** Volcano plot of all genes after pairwise comparison of APOE4/ISO/OP vs. APOE4/Sham (A) and APOE3/ISO/OP vs. APOE3/Sham (B). **C-F** Pathway enrichment analysis and heatmap of up- (C, E) and downregulated (D, F) DEGs derived from the APOE4/ISO/OP vs. APOE4/Sham comparison. **G-J** Pathway enrichment analysis and heatmap of up- (G,I) and downregulated (H, J) DEGs derived from the APOE3/ISO/OP vs. APOE3/Sham. n=4 (APOE4/Sham), 4 (APOE4/ISO/OP), 4 (APOE3/Sham), and 6 (APOE3/ISO/OP).

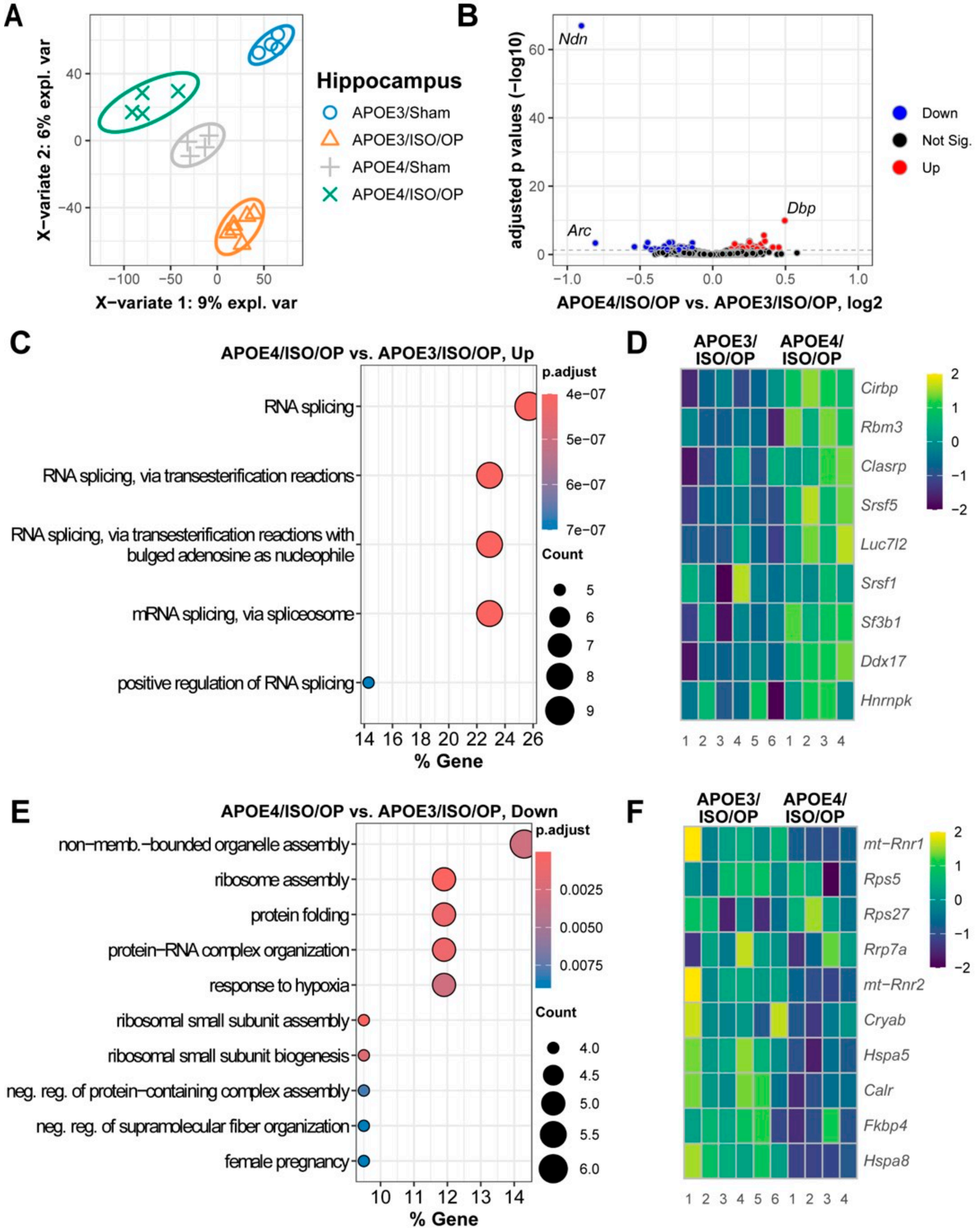
A
Hippocampus
APOE3/Sham
APOE3/ISO/OP
APOE4/Sham
APOE4/ISO/OP
X−variate 1: 9% expl. var
X−variate 2: 6% expl. var
B
Ndn
Arc
Dbp
Down
Not Sig.
Up
adjusted p values (−log10)
APOE4/ISO/OP vs. APOE3/ISO/OP, log2
C
APOE4/ISO/OP vs. APOE3/ISO/OP, Up
RNA splicing
RNA splicing, via transesterification reactions
RNA splicing, via transesterification reactions with bulged adenosine as nucleophile
mRNA splicing, via spliceosome
positive regulation of RNA splicing
p.adjust
Count
% Gene
D
APOE3/ ISO/OP
APOE4/ ISO/OP
Cirbp
Rbm3
Clasrp
Srsf5
Luc7l2
Srsf1
Sf3b1
Ddx17
Hnrnpk
E
APOE4/ISO/OP vs. APOE3/ISO/OP, Down
non−memb.−bounded organelle assembly
ribosome assembly
protein folding
protein−RNA complex organization
response to hypoxia
ribosomal small subunit assembly
ribosomal small subunit biogenesis
neg. reg. of protein−containing complex assembly
neg. reg. of supramolecular fiber organization
female pregnancy
p.adjust
Count
% Gene
F
APOE3/ ISO/OP
APOE4/ ISO/OP
mt−Rnr1
Rps5
Rps27
Rrp7a
mt−Rnr2
Cryab
Hspa5
Calr
Fkbp4
Hspa8

Figure S15 APOE4 mice show distinct transcriptomic signatures in the hippocampus (HI) at 90d following isoflurane (ISO) exposure. **A** PLS-DA plot of normalized gene expression profiles in APOE4 and APOE3 mice under ISO and sham conditions. **B** Volcano plot displaying differentially expressed genes (DEGs) in the APOE4/ISO/OP vs. APOE3/ISO/OP comparison. **C-D** Pathway enrichment analysis of up (C) and downregulated (D) DEGs with Gene Ontology molecular processes. **E-F** Heatmaps displaying DEGs associated with the top three upregulated pathways (E) and top three downregulated pathways (F) identified between APOE4/ISO/OP and APOE3/ISO/OP groups. Heatmaps illustrate sample-wise variation (columns) across genes (rows). Color intensity reflects relative transcript abundance across samples: warmer colors for higher, cooler for lower. n=4 (APOE4/ISO/OP) and 6 (APOE3/ISO/OP) mice.

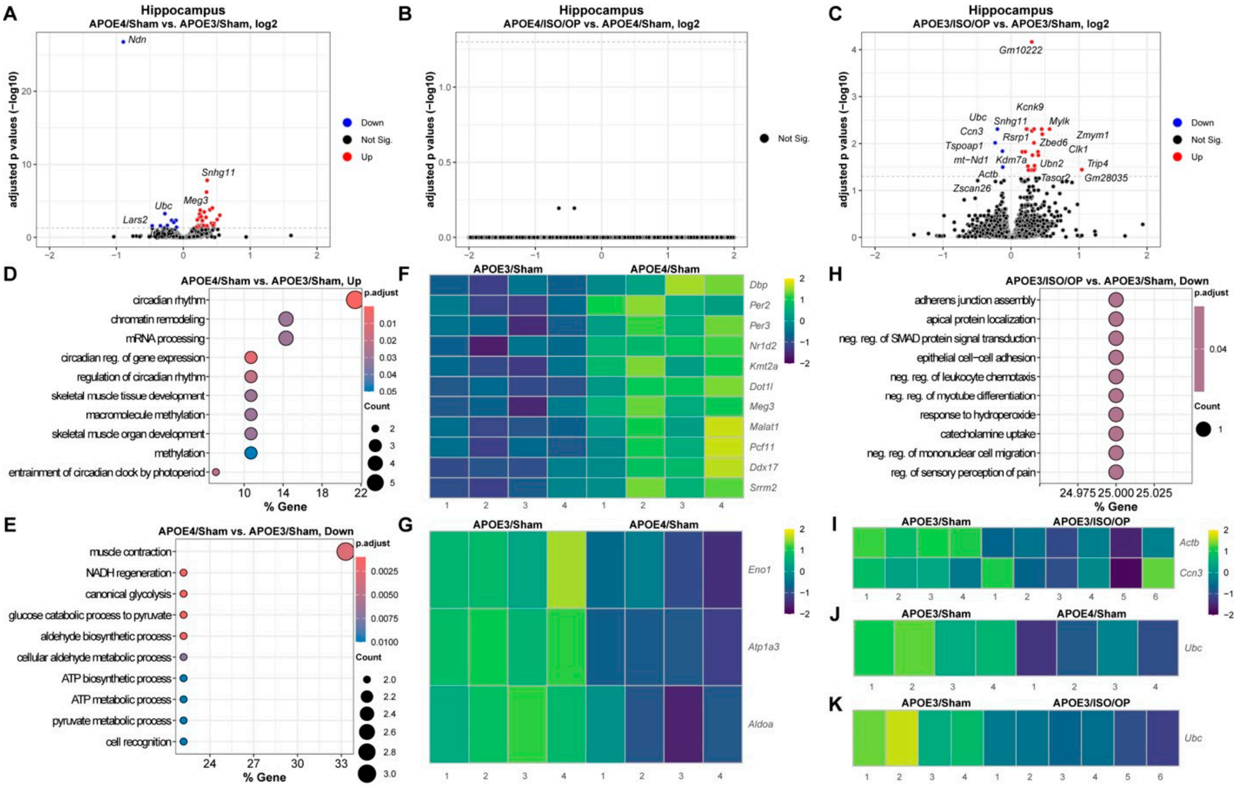

Figure S16 Transcriptomic profiling of the hippocampus (HI) reveals distinct baseline and isoflurane (ISO) induced changes in APOE4 and APOE3 mice 90 days after exposure. **A** Volcano plot of all genes after pairwise comparison of APOE4/Sham vs. APOE3/Sham. **B-E** Pathway enrichment analysis via GO molecular processes of the upregulated (B) and downregulated (D) DEGs and heatmap of genes involved in the top three pathways (C, E). **F** Volcano plot of all genes comparing APOE4/ISO vs. APOE4/Sham. (G) Volcano plot of all genes comparing APOE3/ISO/OP vs. APOE3/Sham. **H-I** GO molecular process enrichment of downregulated DEGs (H), with corresponding heatmaps of representative genes from the top three pathways (I). n=4 (APOE3/Sham), 4 (APOE4/Sham), 4 (APOE3/ISO/OP), and 6 (APOE4/ISO/OP) mice.

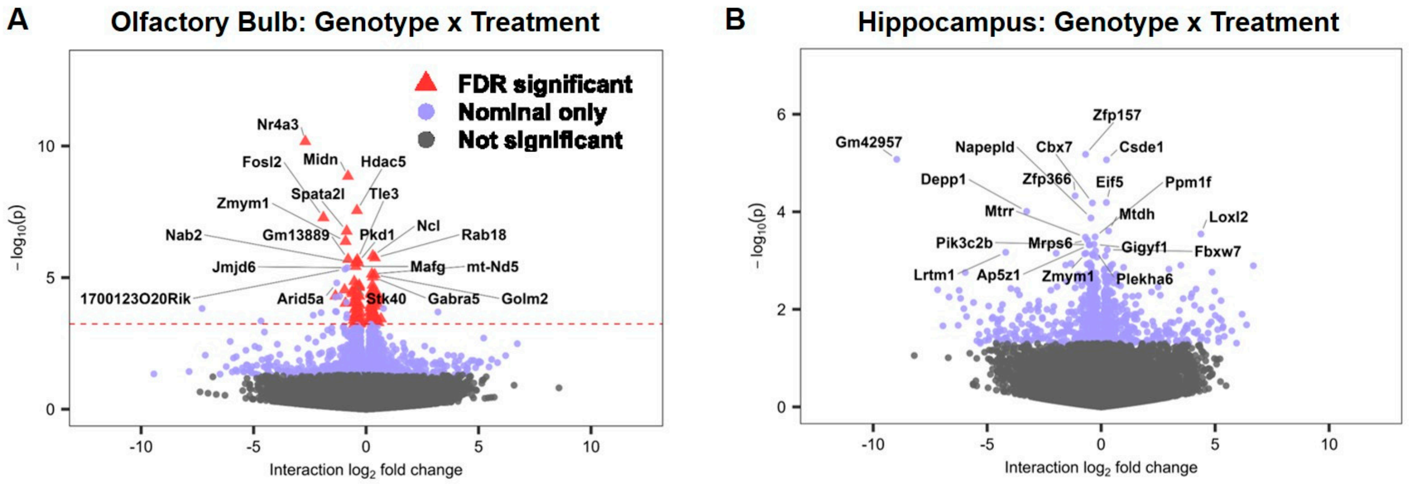

Figure S17 Genotype × treatment interaction analysis of olfactory bulb (OB) and hippocampal (HI) gene expression 90 days after general anesthesia and surgery. Volcano plots show genotype × treatment interactions in the OB (**A**) and HI (**B**), assessed using DESeq2 models including genotype, treatment and their interaction. The x-axis represents the interaction coefficient, calculated as the ISO/OP-associated $\log_2$ fold change in APOE4 mice minus that in APOE3 mice; the y-axis shows $-\log_{10}$ of the nominal interaction Wald-test p-value. Red triangles indicate genes with Benjamini-Hochberg-adjusted $p<0.05$; lavender circles indicate nominal $p<0.05$ without meeting the FDR criterion, including genes excluded from adjusted p-value calculation by independent filtering; grey circles indicate nominal $p\geq0.05$. The 20 genes with the lowest nominal interaction p-values are labelled in each region. The red dashed line in OB marks the observed raw p-value boundary corresponding to FDR significance among genes retained for multiple-testing correction. No corresponding line is shown for HI because no genes met the FDR criterion. There were 99 FDR-significant interaction genes in OB and none in HI. No additional fold-change threshold was applied. Sample sizes per region were n=4 APOE3/Sham, 6 APOE3/ISO/OP, 4 APOE4/Sham, and 4 APOE4/ISO/OP.

Table S1. Coefficients of variation for lipid classes.

Comparison	Region	Cell Type	Group	CV (%)		
				CL	Hex Cer	TG
**APOE4/ISO/OP vs. APOE3/ISO/OP (Figure 1)**	OB	Astrocyte	APOE4/ISO/OP	48.2	52.5	28.9
			APOE3/ISO/OP	19.8	14.3	48.7
	OB	Microglia	APOE4/ISO/OP	41.9	45.4	30.4
			APOE3/ISO/OP	47.3	51.7	48.8
	HI	Astrocyte	APOE4/ISO/OP	41.7	31.4	28.9
			APOE3/ISO/OP	28.8	47.9	29.6
	HI	Microglia	APOE4/ISO/OP	26.0	25.7	22.0
			APOE3/ISO/OP	50.4	45.6	48.6
**APOE4/ISO/OP vs. APOE4/Sham (Figure S2)**	OB	Astrocyte	APOE4/ISO/OP	48.2	58.6	28.7
			APOE4/Sham	29.2	13.5	23.7
	OB	Microglia	APOE4/ISO/OP	40.6	42.0	30.4
			APOE4/Sham	46.1	59.7	36.6
	HI	Astrocyte	APOE4/ISO/OP	34.6	31.4	28.9
			APOE4/Sham	31.9	16.4	43.5
	HI	Microglia	APOE4/ISO/OP	25.8	25.7	22.0
			APOE4/Sham	16.3	12.6	29.3
**APOE3/ISO/OP vs. APOE3/Sham (Figure S3)**	OB	Astrocyte	APOE3/ISO/OP	23.8	31.4	31.7
			APOE3/Sham	18.5	50.6	23.6
	OB	Microglia	APOE3/ISO/OP	31.1	26.6	31.4
			APOE3/Sham	29.7	33.8	22.8
	HI	Astrocyte	APOE3/ISO/OP	29.1	43.9	30.4
			APOE3/Sham	63.5	44.5	33.3
	HI	Microglia	APOE3/ISO/OP	47.6	37.2	25.0
			APOE3/Sham	22.8	15.4	32.8

**APOE4/Sham vs. APOE3/Sham (FigureS4)**	OB	Astrocyte	APOE4/Sham	28.7	13.5	23.7
			APOE3/Sham	40.2	34.7	31.8
	OB	Microglia	APOE4/Sham	45.7	56.8	38.6
			APOE3/Sham	40.1	48.5	34.8
	HI	Astrocyte	APOE4/Sham	32.0	16.4	42.5
			APOE3/Sham	27.7	49.6	48.6
	HI	Microglia	APOE4/Sham	15.8	12.6	29.3
			APOE3/Sham	35.9	22.8	30.3

Coefficients of variation (CVs) for cardiolipin (CL), hexosylceramide (HexCer), and triglyceride (TG) were calculated across three biological replicate samples within each experimental group for astrocytes and microglia isolated from the olfactory bulb (OB) and hippocampus (HI) of APOE3 and APOE4 mice subjected to isoflurane anesthesia and surgery (ISO/OP) or Sham treatment. Comparisons correspond to those presented in Figure 1 and Figures S2-S4. CV values are expressed as percentages. Each biological sample was pooled from three male mice (n=3 samples/9 mice/group).

Table S2. Coefficients of variation of lipid internal standards.

IS CVs (%)	15:0-18:1-d7-PC (IS for CL)	Glucosylceramide-d7 (IS for HexCer)	15:0-18:1-d7-15:0 TG (IS for TG)
HI_Astrocyte_APOE4/ISO/OP	26%	10%	17%
HI Microglia APOE4/ISO/OP	6%	7%	2%
OB Astrocyte APOE4/ISO/OP	19%	6%	3%
OB Microglia APOE4/ISO/OP	6%	6%	1%
HI Astrocyte APOE4/Sham	21%	14%	9%
HI Microglia APOE4/Sham	13%	11%	9%
OB Astrocyte APOE4/Sham	19%	5%	3%
OB Microglia APOE4/Sham	10%	10%	8%
HI Astrocyte APOE3/ISO/OP	39%	1%	7%
HI Microglia APOE3/ISO/OP	13%	13%	4%
OB Astrocyte APOE3/ISO/OP	28%	1%	0%
OB Microglia APOE3/ISO/OP	7%	13%	6%
HI Astrocyte APOE3/Sham	30%	9%	4%
HI Microglia APOE3/Sham	31%	14%	9%
OB Astrocyte APOE3/Sham	22%	7%	6%
OB Microglia APOE3/Sham	18%	8%	4%

Coefficients of variation (CVs) were calculated across experimental groups for three isotope-labelled internal standards (IS) representing cardiolipins (CL), hexosylceramides (HexCer), and triglycerides (TG). CVs were used to assess analytical reproducibility and were generally lower than those of the corresponding endogenous lipid species. CV values are expressed as percentages.

Table S3. Electrophysiological sample distribution across experimental groups.

		APOE3/ISO/OP			APOE3/Sham/OP		APOE4/ISO/OP			APOE4/Sham/OP		
		Mouse 1	Mouse 2	Mouse 3	Mouse 1	Mouse 2	Mouse 1	Mouse 2	Mouse 3	Mouse 1	Mouse 2	**Mouse 3**
Olfactory Bulb	R_m	2	2	2	2	6	3	6	6	3	4	2
	RMP	2	2	2	2	6	3	6	6	3	4	2
	AP	2	3	3	2	6	4	6	6	2	4	2
	sEPSC	1	2	2	2	6	2	2	1	3	4	2
	sIPSC	2	1	1	1	4	1	5	5	2	1	2
Hippocampus	R_m	4	2	3	3	5	3	5	7	4	5	4
	RMP	4	2	3	3	5	3	5	7	4	5	4
	AP	4	2	3	3	5	3	5	7	4	4	4
	sEPSC	4	2	3	3	5	3	5	7	4	4	4
	sIPSC	3	1	3	3	5	2	3	7	4	4	4

Numbers indicate the number of recorded cells obtained from each mouse for the indicated electrophysiological measurements in the olfactory bulb and hippocampus. Rm, membrane resistance; RMP, resting membrane potential; AP, action potential; sEPSC, spontaneous excitatory postsynaptic current; sIPSC, spontaneous inhibitory postsynaptic current.

Table S4. Summary of experimental treatments, sham conditions, and assessment time points across study cohorts.

Experimental cohort	Experimental treatment	Sham condition	Assessment time points
Lipidomics (OB and HI astrocytes and microglia)	Laparotomy (~10 min) under 2% ISO in 100% O_2, followed by continued ISO exposure for a total of 2 h	No anesthesia or surgery; remained in home cage	Day 7
In vivo electrophysiology (awake OB recordings)	Headplate implantation and cranial preparation (d0), followed by recovery and habituation; subsequently received 2 h ISO alone in room air (d12 post headplate), without laparotomy	Identical cranial preparation, recovery and habituation, without subsequent experimental ISO exposure	Days 1 and 7 after ISO or corresponding sham time point
Ex vivo electrophysiology (OB and HI patch clamp)	Laparotomy under 2% ISO in room air, followed by continued ISO exposure for a total of 2 h	Laparotomy under brief ISO in room air, followed by recovery shortly after surgery (Sham/OP)	Day 7
Behavioral assessments	Laparotomy (~10 min) under 2% ISO in 100% O_2, followed by continued ISO exposure for a total of 2 h	No anesthesia or surgery; remained in home cage	Four testing rounds: days 2-7, 10-15, 33-38 and 83-88
RNA sequencing (OB and HI)	Laparotomy (~10 min) under 2% ISO in 100% O_2, followed by continued ISO exposure for a total of 2 h	No anesthesia or surgery; remained in home cage	Day 90, following completion of behavioural testing

All experiments were performed in male humanized APOE3 and APOE4 knock-in mice. Separate cohorts were used for lipidomics, in vivo electrophysiology, and ex vivo electrophysiology. RNA sequencing was performed on tissues collected from the behavioural cohort. Assessment points are expressed relative to the experimental ISO/OP or ISO exposure, except where otherwise specified. The electrophysiological cohorts employed distinct experimental paradigms to accommodate their recording requirements. OB, olfactory bulb; HI, hippocampus; ISO, isoflurane; OP, operation (laparotomy).
